# Generalized valences and oxidation scores for solids

**DOI:** 10.1039/d6sc03835b

**Published:** 2026-09-02

**Authors:** Linda S. Reitz, Peter C. Müller, Richard Dronskowski

**Affiliations:** a Chair of Solid-State and Quantum Chemistry, Institute of Inorganic Chemistry, RWTH Aachen University 52056 Aachen Germany drons@HAL9000.ac.RWTH-aachen.de

## Abstract

Classically defined oxidation numbers (in addition to quantum-chemical population analyses) are regularly used to quantify or at least stick to the ionic notion of molecules and solid-state materials. As such, crystalline NaCl is understood as being composed of Na^+^ and Cl^−^, without any necessity for quantum-chemical calculation. The convenient accessibility of tools such as wave function-based Mulliken and Löwdin as well as density-based Bader charges, however, paves the way towards understanding far more complex cases, at least in principle. While classical oxidation numbers are derived from likewise classical recipes, *i.e.*, assigning bonding as well as non-bonding electrons to the more electronegative atom, one may do something similar given first-principles local wave functions for molecules and solids, thereby defining a convenient quantum-chemical scheme to arrive at *ab initio* valences and *ab initio* oxidation scores. Both concepts are calculated from wave function-based charges and bond orders as provided within the LOBSTER approach transforming plane waves into local-orbital wave functions. The applicability of such *ab initio* valences and *ab initio* oxidation scores is demonstrated from a large number of molecular and solid-state examples, and the prospects as well as challenges, both numerically and conceptionally, are critically discussed.

## Abstract 2

Given first-principles local wave functions projected from plane waves, a convenient quantum-chemical scheme has been derived to arrive at *ab initio* valences and *ab initio* oxidation scores for both molecules and solid-state materials.

## Graphical abstract

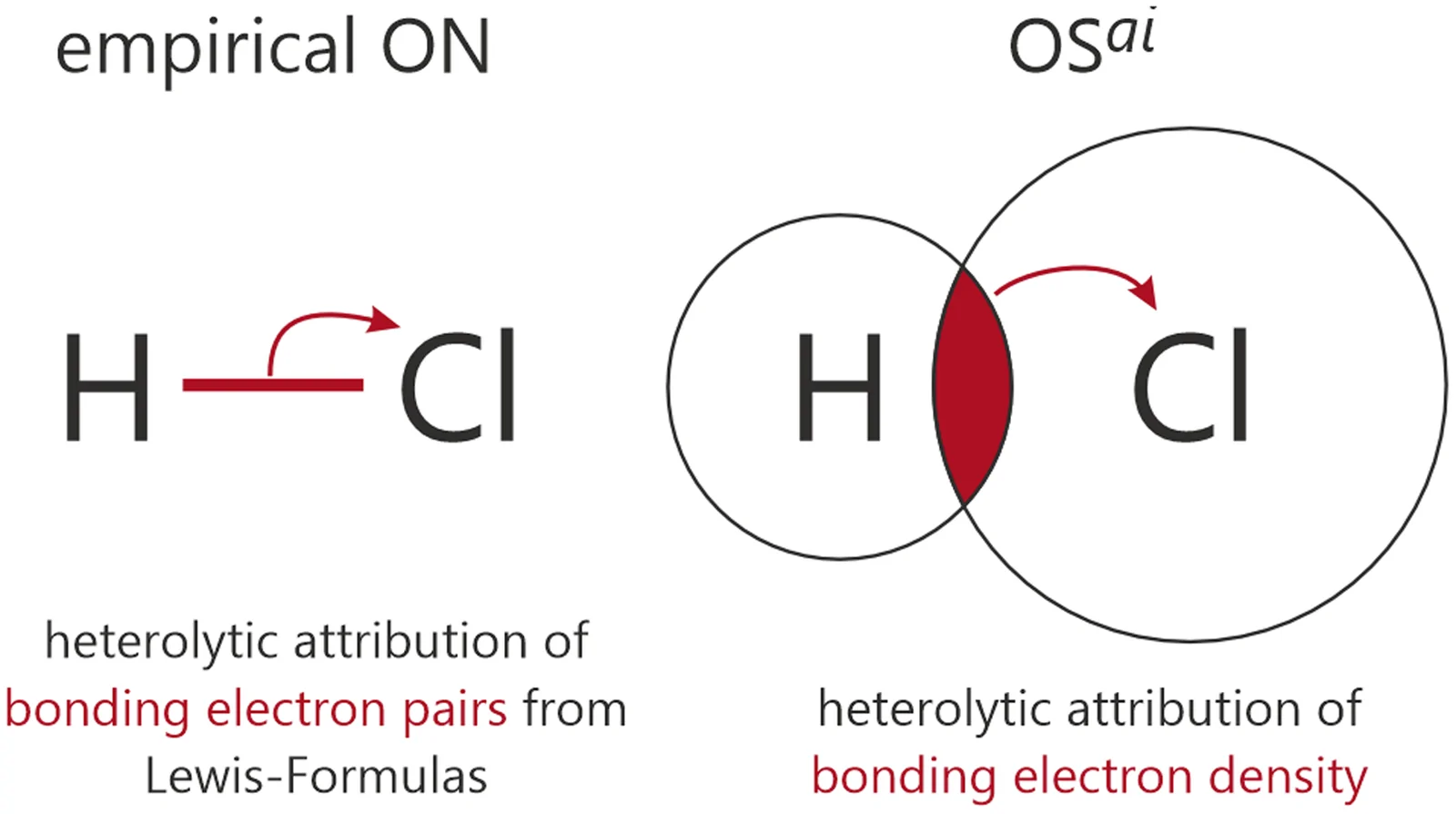

## Introduction

The concept of the oxidation state or number is taught early on in any chemistry curriculum. Various but consistent ways to define it can be found in every basic chemistry textbook, all of them arriving at essentially the same result. The oxidation-number concept, of decisive importance when it comes to balancing redox equations,^1,2^ is also associated with the central atom in a coordination entity, in this case being the charge borne by the central atom if all the ligands were removed along with the electron pairs shared with the central atom.^1,3^ In that spirit, the IUPAC defines the oxidation state of an atom as the charge of this atom after ionic approximation of its heteronuclear bonds.^4^ This means that homonuclear bonds are divided equally while in heteronuclear bonds the electrons are assigned to the more electronegative contributor. To perform this assignment, Allen^5^ electronegativities given in Fig. 1 may be used, as also suggested by the IUPAC, because they are based on the average valence-electron energy of the free atom.^5–7^

**Fig. 1:**
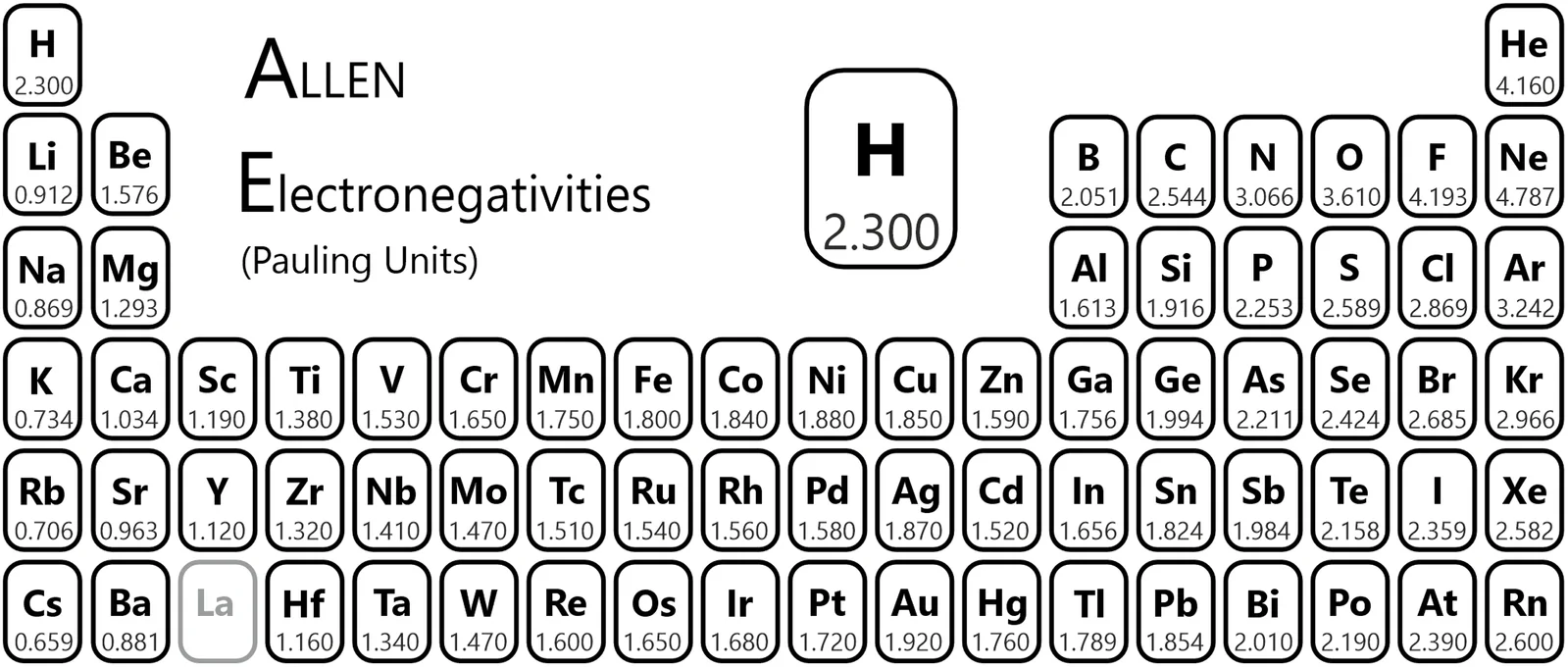
Allen electronegativities^1,5–7^ in Pauling units. Lanthanum is shown in light grey since the lanthanide series has not been evaluated.

Along with the aforementioned definition, two algorithms for allotting an oxidation number (ON) should be mentioned. These are the algorithm of assigning bonds and the algorithm of summing bond orders. The first algorithm is tailored to Lewis structures showing all valence electrons in a molecule, the textbook approach. Here, the heteronuclear bonds are assigned to the more electronegative partner††For matters of completeness we note that exceptions to this assignment are reversibly bonded Lewis-acid ligands, *e.g.*, transition-metal complexes with Z ligands. For more literature on this topic we recommend the decisive contributions by the IUPAC taskforce, *i.e.*, ref. 1, 4, 9 and 12. They cover and also explain this and many other cases in much greater detail than we could have done in this article. identified by an ionic approximation, while homonuclear bonds are divided equally, as mentioned before. The ON then equals the resulting atomic charge.^1,4^ This is demonstrated for H_2_O_2_ in Fig. 2 (left) where the heteronuclear O–H bond is assigned to the more electronegative O atom and the homonuclear bond is divided equally, hence arriving at an ON of +I for H and −I for O.

**Fig. 2:**
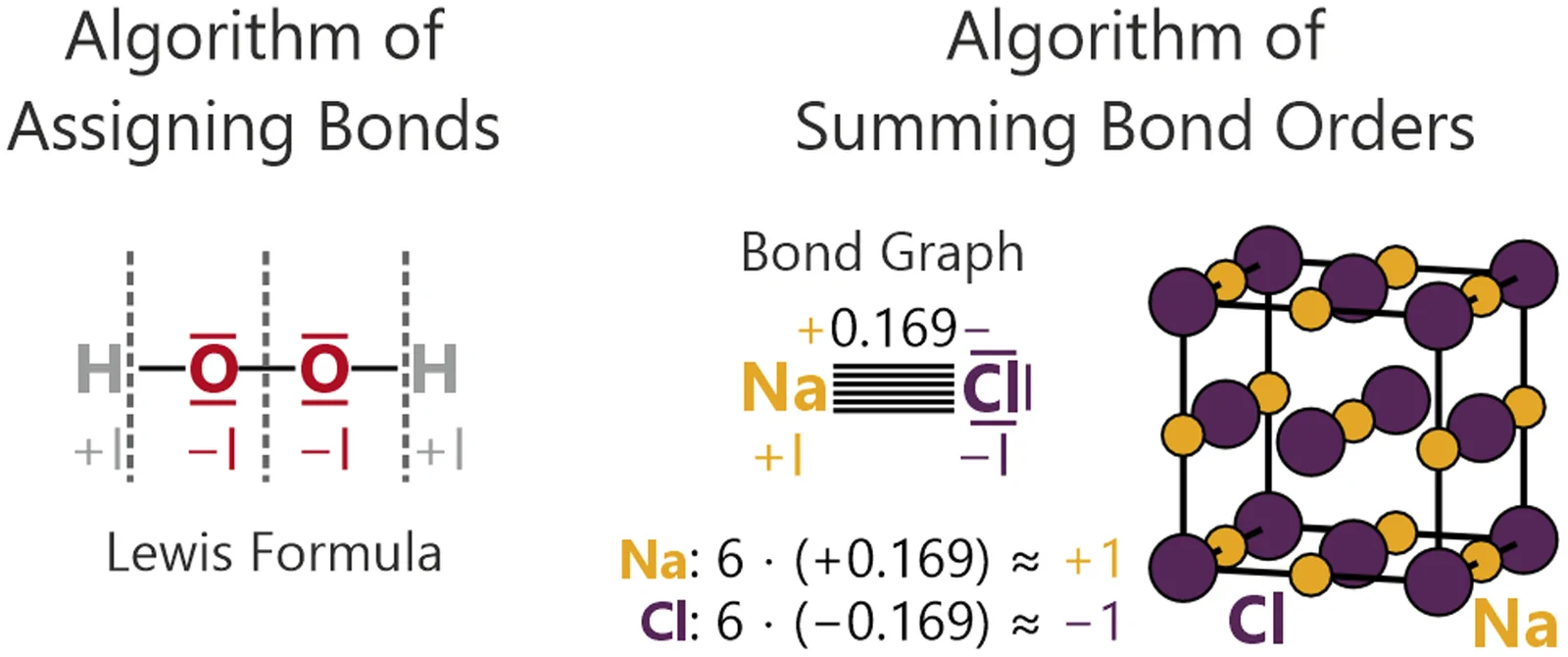
Oxidation numbers obtained using the algorithm of assigning bonds for H_2_O_2_ and the algorithm of summing bond orders for NaCl.

The second algorithm works on Lewis formulae as well as bond graphs, in particular for extended solids. The ON is obtained by summing the heteronuclear-bond orders at each atom as positive/negative if that atom is the less/more electronegative partner. If there is any formal charge to consider for the corresponding atom, it needs to be added to that sum. In the NaCl example given in Fig. 2 (right), a bond graph is drawn, representing the six “ionic” bonds formed by each Na and Cl atom to their nearest neighbouring atoms. If we then rely on the bond-valence concept^8^ and empirically tabulated data (*r*_0_ = 2.15 Å) for the Na–Cl combination, a bond order of approximately 0.169 is obtained for each bond, so the ON is the sum of the heteronuclear bond orders at each atom (6 × 0.169 = 1.014 ≈ 1) after weighing them based on their ionic sign at that atom.^1,4^ This leads to an ON of +I for Na and −I for Cl.

Apart from these algorithms, we provide a set of rules or classical instructions on the oxidation-state or oxidation-number assignment, including textbook recipes that can be derived from the definition of the ON, perfectly valid by definition, taught and widely used today.^1,4,9^ They have been gathered from a few textbooks and articles,^1,2,10,11^ and the order is arbitrary. As we will touch upon in the following sections, they look like this:

(1) The oxidation number of an atom in the elementary state is zero.

(2) In a compound, the oxidation number of the element with the lower/higher electronegativity is positive/negative.

(3) The algebraic sum of the oxidation numbers of atoms in a compound is zero.

(4) Alkaline metals in compounds have the oxidation state +I, and alkaline-earth metals +II.

(5) The oxidation number of hydrogen in compounds is +I with the exception of metal hydrides where it is −I.

(6) The oxidation number of oxygen in compounds is −II. Exceptions to this rule are peroxides where it is −I and fluorides, *e.g.*, OF_2_, where it is +II.

(7) Fluorine in a compound has the oxidation state −I.

For completeness, we should add that oxidation numbers do not have to be an integer, in perfect harmony with the IUPAC, even though this impression can be found in some textbooks, primarily for didactical purposes. As a typical example, the oxidation number is −0.5 in the O_2_^−^ hyperoxide anion. In the N_3_^−^ azide anion the ON is widely known to be −0.333. Alternatively, an IUPAC technical report from 2014 (ref. 12) formulates the azide anion either with an ON of −0.5 or −1 on the terminal N atoms and a neutral (*i.e.*, N^−0.5^ N^0^N^−0.5^) or cationic N at the center (*i.e.*, N^−1^ N^+1^N^−1^), both expressions differing in their respective bond orders. The average ON of −0.333 is then recommended as suitable for electrochemical purposes. Either way, the IUPAC also uses fractional oxidation numbers, sometimes yielding differing solutions. In other words, such oxidation numbers are classically derived or, more correctly, *defined*, so they do not necessarily model the electronic structure of a given molecule or solid. Instead, they cleverly keep track of all involved electrons, reshuffling them as a function of the electronegativity difference between atoms, at the same time keeping in mind important principles of main-group chemistry. We also note that oxidation numbers are here and there (unfortunately) called “formal” charges, in particular in theoretical physics, but we refrain from using that term since it may lead to confusion. It is rather obvious that all such idealized charges are formal, by sheer definition, but formal charges are something entirely different, derived from *homolytic* bond splitting in all cases, as given in the IUPAC definition.^13^ Because of that, the oxidation numbers in the CO molecule are +II for C and −II for O whereas the formal charges are ⊖ for C and ⊕ for O, the circles highlighting the formality. Quite surprisingly, the latter formal-charge concept provides the correct sign for the dipole moment of carbon monoxide, unlike the electronegativity.

Coming back to oxidation numbers, we are not aware of any fundamentally difficult molecular cases but there are highly complex bonding situations, like those prevailing in solid state structures, where the assignment of the ON is still ambiguous, despite proper rules: if the electronic structure gets complex, classical oxidation numbers may become problematic too. A prominent example is the ternary metallic nitride Ce_2_MnN_3_, where the (Ce^+III^)_2_Mn^+III^(N^−III^)_3_ formulation was proposed based on redox potentials from aqueous solution. Alternatively, the ionic notion (Ce^+IV^)_2_Mn^+I^(N^−III^)_3_ is also possible, and magnetic data do not allow low-spin Mn^+III^ and low-spin Mn^+I^ to be distinguished. This problem was eventually solved by electron-density and also COHP analysis obtained using TB-LMTO-ASA, yielding an ON of +IV for Ce to be the more reasonable choice, surprisingly enough.^14^ In other words, Ce_2_MnN_3_ is not mixed-valent as regards cerium or manganese.

A quarter of a decade later and given far more powerful tools, the interest in a computational approach is evident, as demonstrated by various ones described across the literature. As we are especially interested in first-principles techniques, let us mention one population-analytic method based on localized molecular orbitals and using a threshold to assign the electrons to the respective atoms.^15^ Another approach employs projection techniques to obtain the central atom's d-orbital populations in a transition-metal complex.^16,17^ There is also the so-called effective oxidation state (EOS) analysis which can also be applied to the solid state^18^ and accommodate different definitions of atoms in molecules. It relies on spin-resolved effective atomic orbitals (eff-AOs) which are sorted by occupation number. So-called α electrons are assigned to the eff-AOs of the centres with higher occupation numbers until the number of α electrons is reached, and then everything is repeated for the β electrons.^3,18,19^ Yet another method uses wave-function topology and polarization theory to assign electrons to atoms.^20^ It exploits the change in polarization during ionic transport that is directly translated into the integer oxidation numbers of each considered atom. The carefully selected examples illustrate this theory's applicability to simple molecules and solids as well as more involved solids that contain more than one crystallographically independent site with different oxidation numbers. While this theory stands out due to its astonishing results given proper parametrization, it has not been adopted in the literature. We believe the reason for that to lie either in the large computational effort of the method that requires the calculation of migration paths for each atom, or in parametrization problems (+*U* for main-group elements too), or in the fact that it can only be applied to insulators—polarization is only meaningful if no electrical conductivity is present. Instead, we identify the true demand for such computational approaches to rather lie elsewhere, *i.e.*, in investigating increasingly complex metallic compounds. Apart from a few exceptions, oxidation number assignment in metals is not unambiguous but a quest often found across the literature where common computational tools fail.

In this work we propose an alternative approach to calculate *ab initio* oxidation scores and valences, targeting solids (insulators, semiconductors, and metals) but also molecules. Both are derived from charges and crystal orbital bond indices as implemented in the LOBSTER program. Using these quantities, we desire to mirror the intelligible heterolytic bond splitting used in the empirical calculus of oxidation numbers by using quantum-mechanically derived descriptors. Fig. 3 straightforwardly depicts the conceptional parallel. For a hydrogen chloride molecule, we would naturally draw a single bond between both atoms. As already alluded to before, the oxidation numbers are then determined by heterolytically splitting the bond and assigning all bonding electrons to the more electronegative partner, *i.e.*, chlorine. The quantum-mechanical analogue is readily shown by a Mulliken-style population analysis that separates the total electron density into net and overlap populations. Since the latter is already correlated with the H–Cl bond, we can analogously set up a quantum-chemical charge allocation by assigning the overlap (=bonding) density to chlorine.^21^ The so-derived quantity will be called the *ab initio* oxidation score OS^ai^ to express that there are certain parallels to the classical oxidation number as regards the idea behind allocating bonding electrons. It also needs to be said that whatever the *ab initio* oxidation score OS^ai^ is not intended to challenge (or exactly match) the classical ON even though OS^ai^ are truly needed: so far, partial charges are widely used, sometimes questionably, to describe more complex systems (such as metals) in terms of the oxidation number. With the OS^ai^ introduced, in the following section we wish to provide the community with an *ab initio* concept allowing for a charge assignment closer to what we would expect from the classical oxidation number. Hence, the charge allocation is performed *via* a weighting scheme that uses the difference in orbital energy between the orbitals on two atoms smoothed by an error function. Such an approach is computationally efficient (<5 s for any system shown in this paper) and has therefore been added to the new LOBSTER default output.

**Fig. 3:**
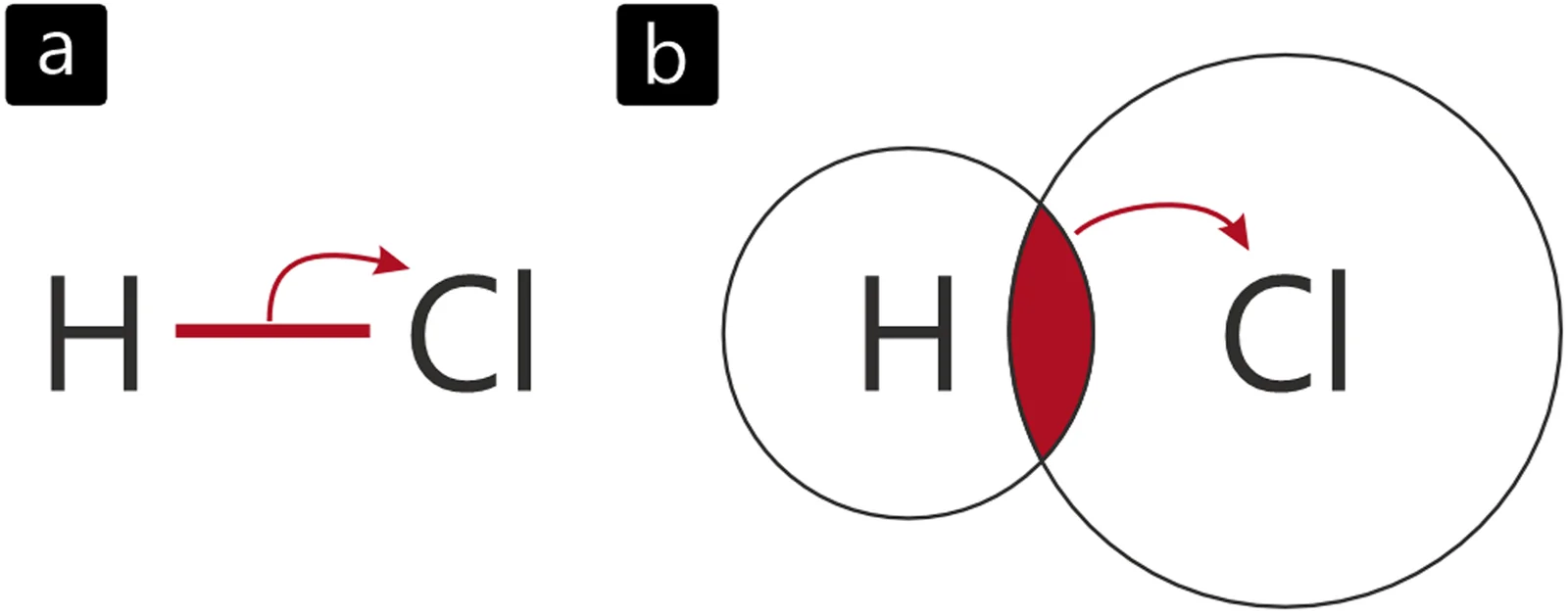
Comparison of the transfer of bonding electrons in an HCl molecule for (a) shifting entire electron-pair bonds on purpose and (b) assignment of electron density in terms of overlap populations. Bonding electrons/overlap population is shown in red. Note the schematic character of the two sketches: shown are pairs of electrons, irrespective of whether or not the calculational scheme operating on the wave function would be Mulliken-like (finite overlap) or Löwdin-like (zero overlap).

## Theory

### Valence

Before calculating the oxidation score (within the ionic limit) itself, we first need to focus on the covalent limit, equivalent to the valence of an atom. According to the IUPAC, the valence is defined as the number of (single) covalent bonds an atom forms,^22^ and it can also be understood as the covalent bond capacity of this atom.

Within the language of quantum chemistry, the *ab initio* valence *V*_A_ of an atom A is given by the sum of the Wiberg bond indices *W* originating from this atom with its neighbors,^23^ following from the idempotency of the density matrix for an orthonormal basis set.^24,25^1
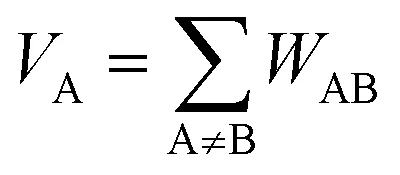
In the sum, A ≠ B ensures that the atom does not bond with itself. For methane, four single bonds (*W*_AB_ = 1) to H then result in a carbon valence *V*_A_ = 4, as expected.

Since the Wiberg bond index has already been generalized to periodic systems by means of the Crystal Orbital Bond Index^26^ (COBI) and its ICOBI energy integral, the valence can analogously be defined as2
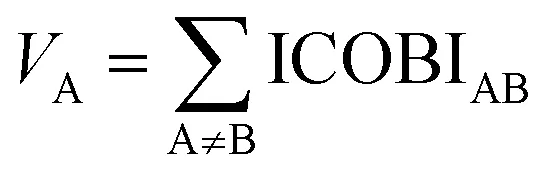


As this sum (theoretically) involves all atom pairs in an infinitely large solid (*i.e.*, a crystal), reciprocal-space calculation drastically improves performance, so *k*-space integration yields3
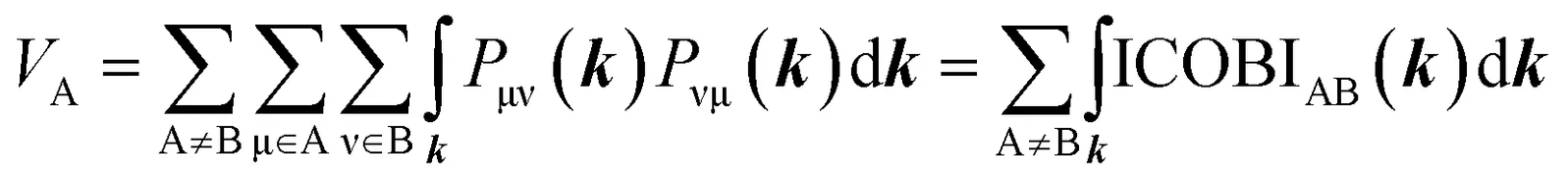
with the density matrix elements *P*_µν_ and *P*_νµ_ for the respective orbital indices µ and ν.

Analogous to the energy-dependent COBI, we can also formulate the valence as a “density of valence” DOV, hence also introducing energy-dependence. By doing so, DOV closely resembles the well-known density of states (DOS) but only includes density from interacting (*i.e.*, between atoms) orbitals.4
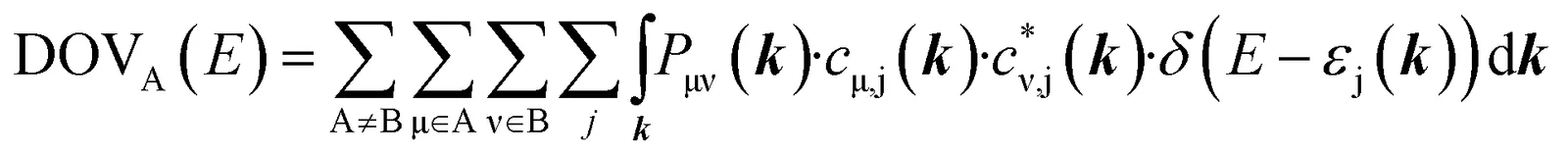


For illustration, a DOV plot including its integrated value (IDOV) is given exemplarily for carbon in the diamond structure in Fig. 4. For carbon, a valence of 4 (*i.e.*, tetravalency) is expected.

**Fig. 4:**
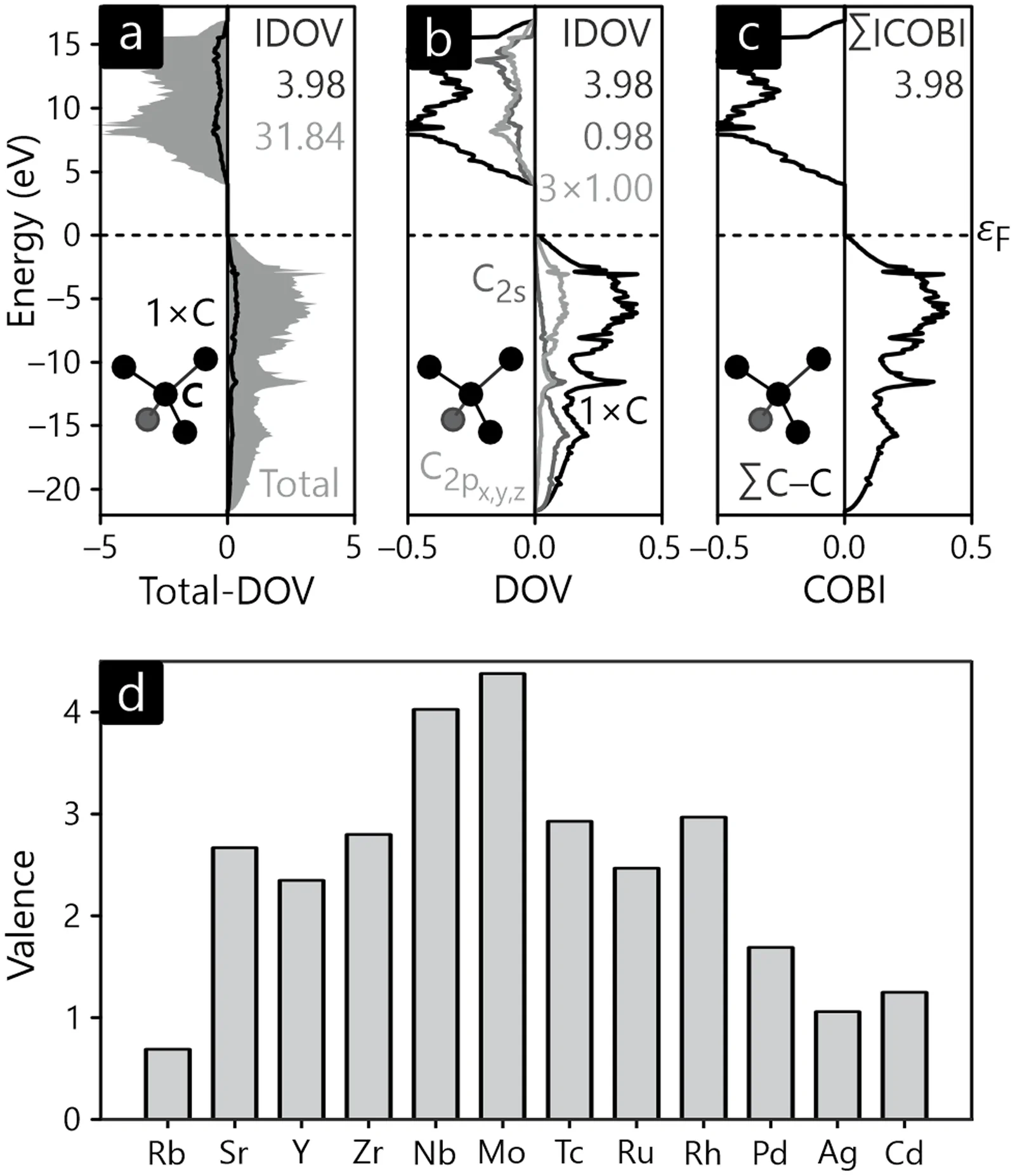
(a) Plot of the total Density of Valence (DOV) for carbon in the diamond structure. The total-DOV is shown in light grey while the DOV of a single contributing carbon atom is depicted in black. The integrated values (IDOV) correspond to the valence (3.98 ≈ 4). The total-IDOV (31.84 ≈ 32) is the sum of all valences in the system. (b) DOV of one carbon atom shown in black with contributions from the 2s and 2_px,y,z_ orbitals. (c) Summed COBI of all bonds one carbon atom forms. (d) Course of the valences from elemental rubidium to cadmium.

The IDOV = 3.98 closely matches the expected valence (4), an encouraging sign. The total IDOV (31.84) in (a) equals the sum of all valences of all the C atoms (eight) in the unit cell. As the DOV is calculated based on the COBI, it is quite intuitive that the shape of the DOV for a single C atom given in Fig. 4(b) equals that of the COBI, as shown in Fig. 4(c), of all bonds this atom forms. The same is observed for the integrated values of both descriptors. The DOV and valence can also be looked at in an orbital-resolved manner, as shown in Fig. 4(b). In the case shown here, the 2s orbital on C has a valence of 0.98 while each 2p orbital on C has a valence of 1.00. Because the contribution of 2s and 2p is essentially the same, the simplified “sp^3^” valence-bond notion is nicely justified from density-functional theory. It is fairly obvious that the valence is a consequence of the number of available valence electrons, most easily seen from the main-group elements (4 for carbon, 5 for nitrogen, *etc.*). To demonstrate this idea given a more elaborate example, let us investigate the valence of the 5s4d elemental metals in the fifth period from rubidium to cadmium shown in Fig. 4(d), a simple example avoiding problems with spin polarization (seen for the 3d elements) or relativity (seen for the 5d elements).

The course of the plot is in agreement with our expectation, roughly measuring the number of valence electrons involved. For rubidium a valence of approximately 0.7 is found, a little smaller than the single valence electron in the 5s orbital. In Sr the valence is higher than the two expected valence electrons because a partial population of the 4d orbitals is in reach energetically, all taking part in the chemical bond. Moving on to Y, one might anticipate a lower valence by assuming a 5s^2^4d^1^ electronic configuration but the gross orbital population yields that 5s is only occupied by one electron and the 4d orbitals more populated, and this results in an increased valence of 2.4. As already described by Schwarz in 2010,^27^ the reason lies in the d-orbital collapse which results from an interplay of nuclear attraction as well as imperfect shielding by the inner-core electrons and angular-momentum dependent centrifugal forces.

After Y, the valence increases with the d-electron count until it reaches its maximum for Mo. The large valence of 4.4 mirrors, at least to some degree, the anticipated electronic configuration of 5s^1^4d^5^. After this maximum, the course of the valence declines, inversely mirroring the previous path. Clearly, the number of available valence electrons causes that behaviour, also alluding to the maximally reachable oxidation numbers known from the chemistry of those metals.

### Oxidation numbers

Having defined the *ab initio* valence, it is rather straightforward to calculate *ab initio* oxidation scores by strictly assigning the bonding electrons—quantified by the ICOBI—to the more electronegative bonding partner, much like in the classical recipe. To do so, we will exploit a key property of the density matrix in an orthonormal basis, namely the idempotency, *i.e.*,5
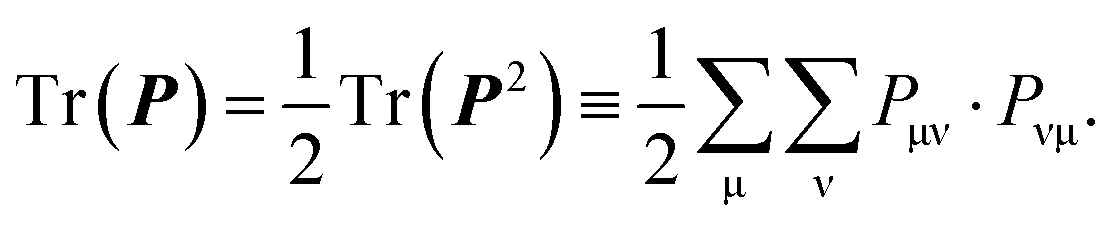


This simply allows an orbital's gross population *P*_µ_ to be derived by means of the density matrix elements *P*_µν_*via*6
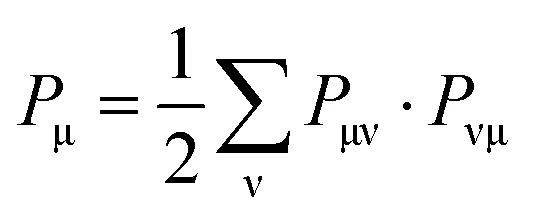


Notably, the density matrix shown in eqn (5) and (6) symmetrically attributes the bonding density to both participating atoms, similar to Mulliken's population analysis.^28,29^ For further reference, we included a pictorial derivation of this assignment by a heat map shown in Fig. S1. The homolytic splitting, however, does not reflect differences in electronegativity or bond polarization which would require a heterolytical bond splitting instead. With this in mind, we now introduce a weighting factor *w*_µν_ and define the *ab initio* oxidation score of atom A (OS^ai^_A)_ as7
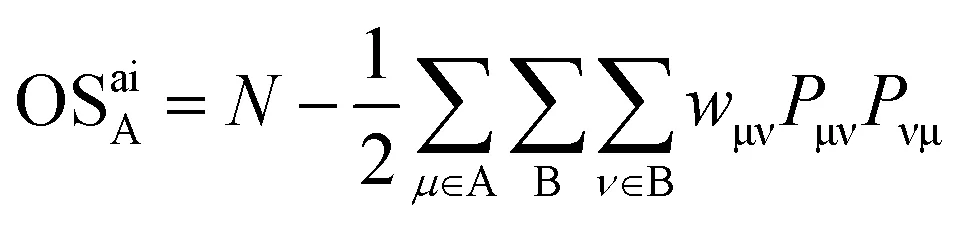


This weighting factor is needed to incorporate a maximally ionic assignment of the bonding electrons upon heterolytic bond splitting, reflecting the oxidation-number recipe. The weighting factor is determined based on the energetic difference between the orbitals µ and ν, so using Hamilton matrix elements *H*_µµ_ might seem to be the logical choice, as described in our previous work.^30^ We perform, however, a unitary transformation of the electronic structure from a plane-wave basis to an atomic-orbital picture using LOBSTER. As this procedure relies on density-functional theory, the projected atomic-orbital eigenvalues are affected by DFT-typical issues, say, underestimation of band gaps given some particular exchange–correlation functional. Consequently, the orbital order may change depending on the quality of the band gap, which may lead to ambiguity in the long run. This is why, in the present work, we select the *density matrix* elements *P*_µµ_ that have already been used before^21^ to weight the overlap population in Mulliken's population analysis. The analogy between Hamilton and density matrix results from the *aufbau* principle: as orbitals are filled with increasing energy, an orbital with full occupation will have a lower energy compared to a partially occupied orbital, but the main advantage of the density matrix is that conduction bands are unambiguously defined by having zero occupation. The weighting factor is then formulated as follows:

8 *w*_µν_ = 1 + erf(*α*(*P*_µµ_ − *P*_νν_))

The parameter *α* determines the smoothness of the splitting algorithm between the orbitals µ and ν. Fig. 5 shows *w*_µν_ for different values of *α*. For empirical oxidation numbers, there is a hard cut between the atoms as the more electronegative atom takes all bonding electrons.

**Fig. 5:**
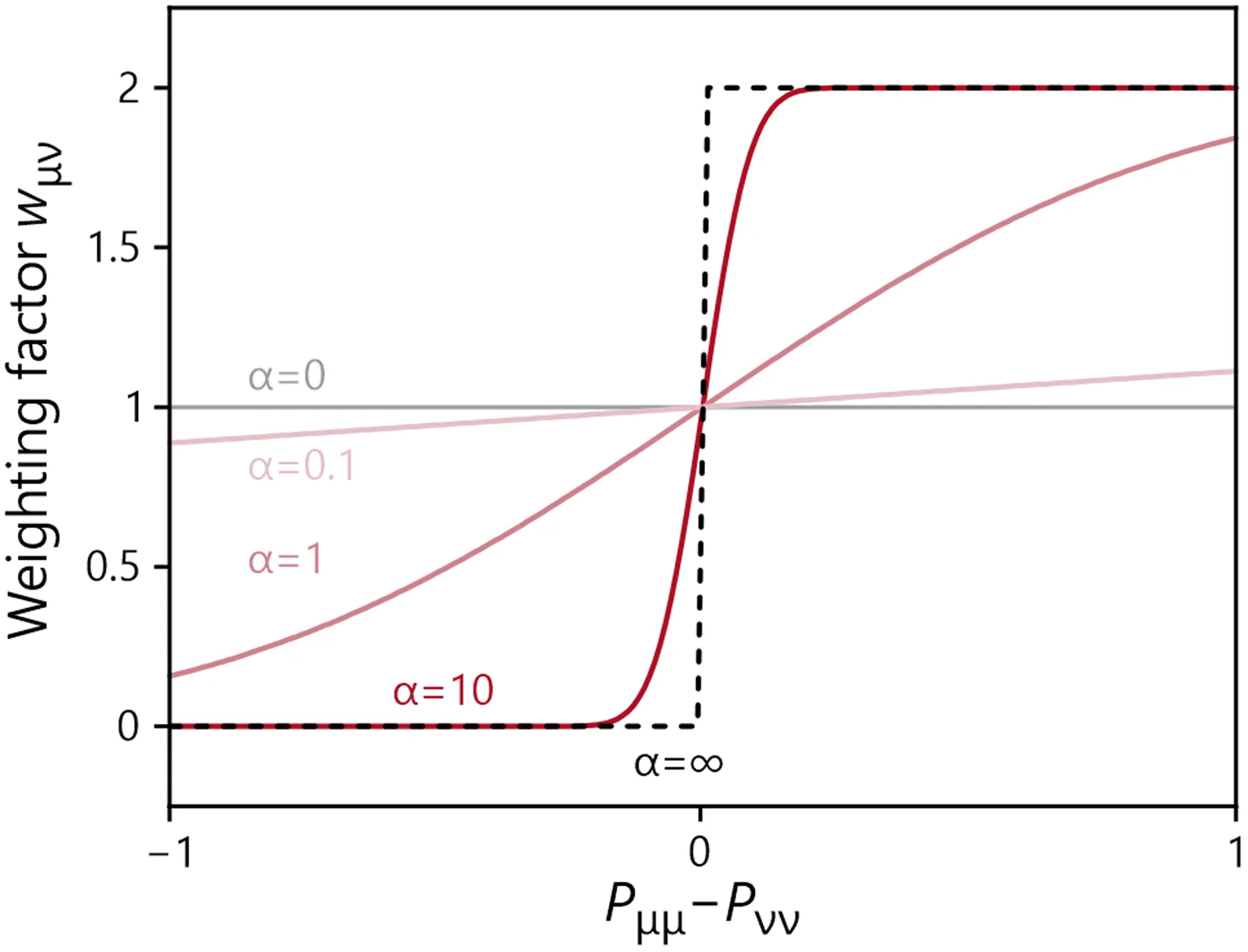
Weighting factor for different values of *α*.

For the *ab initio* oxidation scores presented here, this corresponds to an infinitely large *α* (black dashed curve in Fig. 5). As long as *P*_µµ_ is smaller than *P*_νν_, all electrons go to orbital ν as the weight is zero. If both are equal, *i.e.*, *w*_µν_ = 1, the splitting is symmetric, and as soon as *P*_µµ_ increases, *w*_µν_ = 2. Such a drastic partitioning, however, can overestimate the polarization of a bond if the orbitals µ and ν are close in energy, and similarly, numerical differences in the density matrix can cause large errors. If *α* is chosen to have a finite value (red curves in Fig. 5), the smoothness of the partitioning can be controlled from fairly hard (*α* = 10, red curve) to fairly smooth (*α* = 0.1, light red curve). If *α* is chosen to be 0, the weighting factor is always 1 and the resulting oxidation score OS^ai^ equals the corresponding Löwdin charge.

We note that the entire theoretical procedure leading to the density matrix is from first principles, purely *ab initio*, without empirical parameters. To arrive at the chemists' arbitrary heterolytical bond splitting as defined by the oxidation-number concept, a likewise arbitrary *α* parameter cannot be avoided such as to not cause unreasonable shifts of bonding electrons. Let us explain how to shift bonding electrons in more detail. Eqn (9) shows the general calculation of the oxidation number for Li in molecular LiI:9
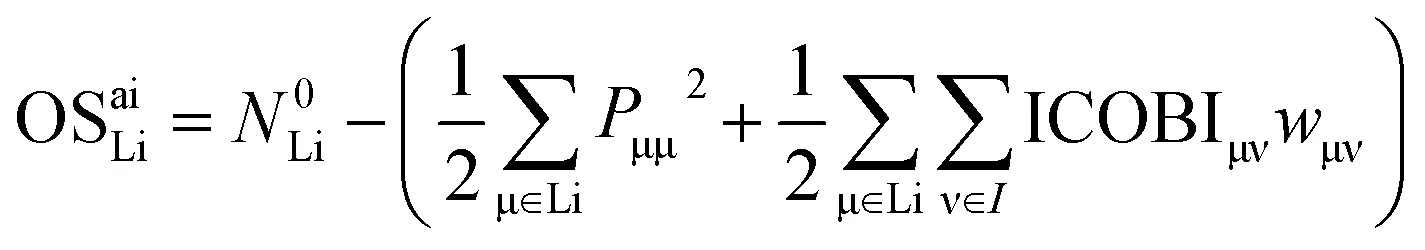
Here N^0^_Li_ is the valence-electron number in the neutral atom (1 for 2s^1^, also in the pseudopotential used here), *P*_µµ_ is the density-matrix element of orbital µ, and ICOBI_µν_*w*_µν_ corresponds to the shifted bonding electrons of the Li–I bond.

For better visualization, we present the numerical details for the two limiting cases of homolytic and heterolytic bond splitting in Fig. 6a and b. The homolytic assignment (*α* = 0) in Fig. 6(a) pushes half of the bonding electrons within both relevant interactions to each contributing atomic orbital. In the case of the 2s–5p_z_ interaction, a total of 0.22 bonding electrons is homolytically redistributed, so both 2s and 5p_z_ are assigned 0.11 electrons each. In case of a heterolytic splitting (*α* ≫ 0), the total 0.22 bonding electrons are fully assigned to the I–5p_z_ orbital and none to Li-2s. In total, we arrive at Löwdin populations of 0.20 for Li and 7.80 for I as well as weighted populations of 0.02 for Li and 7.98. The resulting oxidation numbers of ±0.98 match the empirical integer oxidation numbers whereas the Löwdin charges of ±0.80 are significantly smaller. As alluded to already, the choice of *α* is arbitrary but it should mirror the same relation as the empirical oxidation numbers, as shown in Fig. 6. Therefore, we chose *α* = 10 for all subsequent analyses as this value seems practical and robust too.‡‡The *α* parameter cannot be used to select a “better” result, it merely controls the harshness of the assignment of bonding electrons. More details on the choice of *α* and its robustness are given alongside Fig. S2 in the SI.

**Fig. 6:**
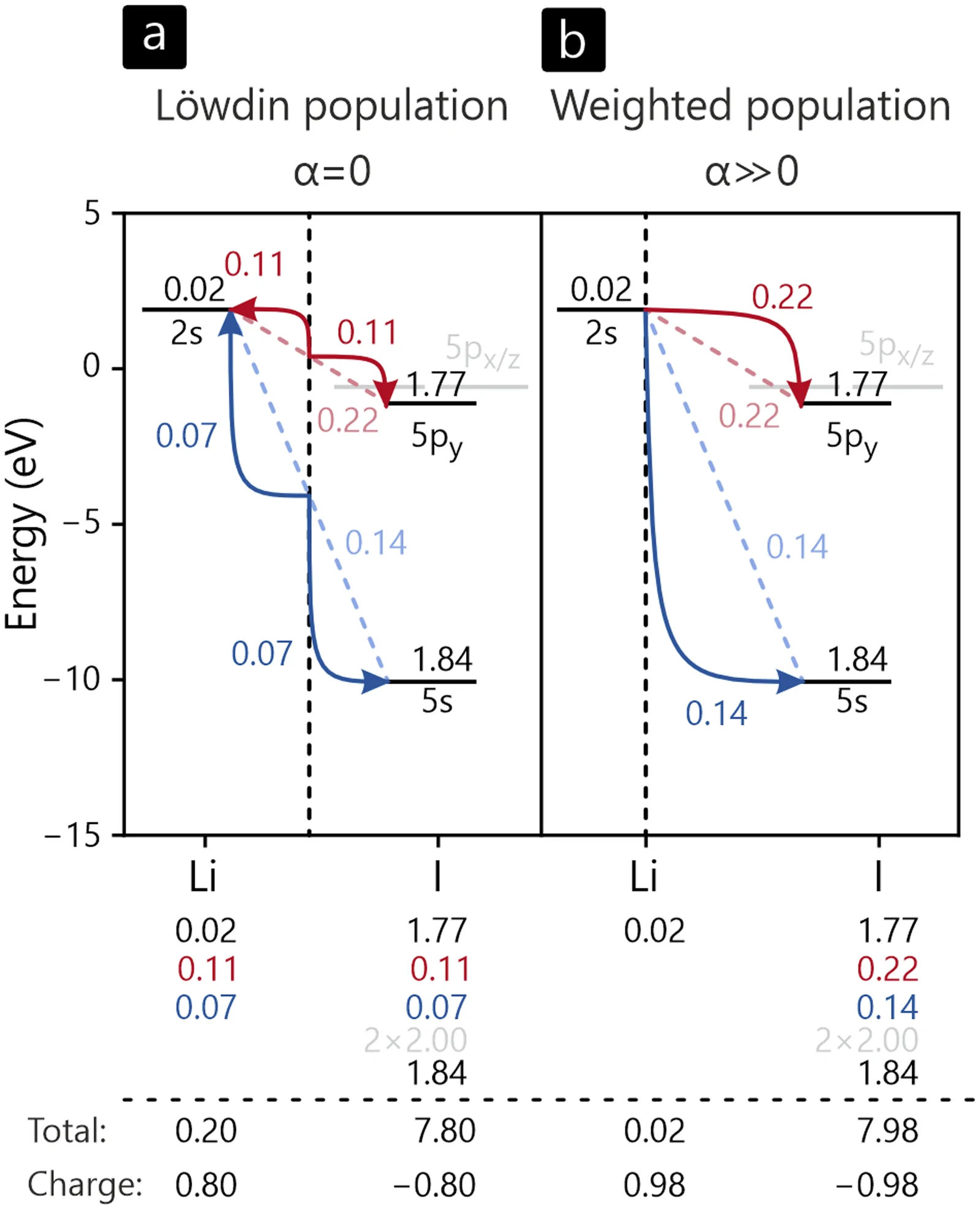
Assignment of electrons for two limiting cases of the weighting factor. (a) *α* = 0 corresponds to the Löwdin population analysis. (b) *α* ≫ 0 yields the *ab initio* oxidation scores.

As written in the introduction, the electronegativity difference is commonly used to quickly estimate the ionicity of a chemical bond. In particular, Pauling correlated the ionicity obtained from a molecule's dipole moment with ΔEN for different single-bonded molecules in the gas phase.^31^ In the same spirit, we calculated the *ab initio* oxidation scores for selected molecular alkali halides, hydrogen halides and several interhalogen compounds, as given in Fig. 7a. The thus derived oxidation scores of these molecules are almost perfectly integer ±1 for all alkali halides—from the strongly ionic CsF to the rather covalent LiI. For electronegativity differences below 1.0, however, we find fractional OS^ai^ for polar covalent molecules such as HCl, HBr and ICl. Interestingly, almost all selected compounds lie perfectly on the fitted curve shown in red, and only HF is slightly off the mark compared to compounds with similar ΔEN, *e.g.*, KBr.

**Fig. 7:**
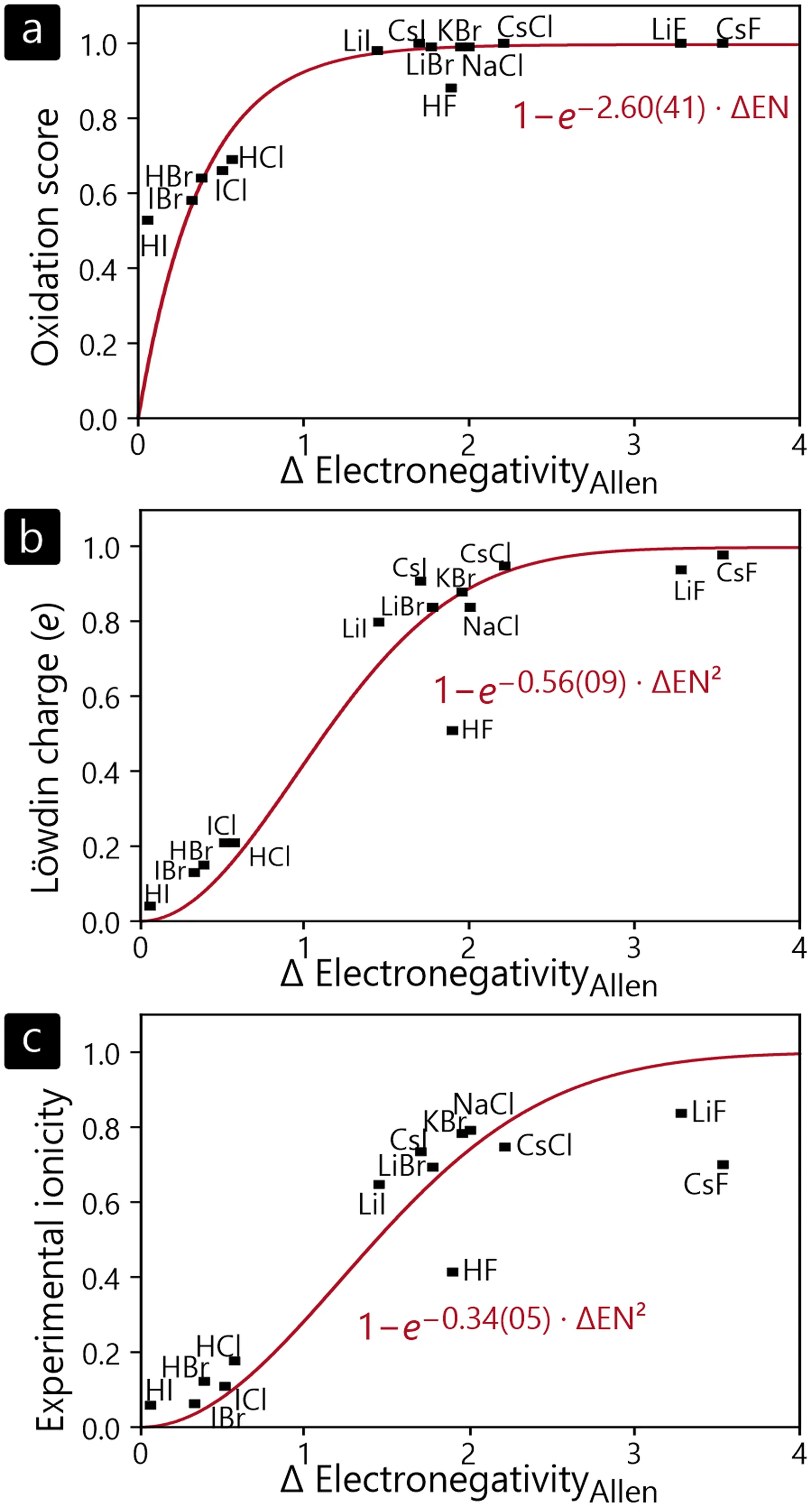
(a) *ab initio* oxidation score OS^ai^, (b) Löwdin charges and (c) experimental ionicity for selected diatomic molecules involving single bonds. Fitted curves for all correlations are given in red.

Admittedly, non-integer oxidation scores and numbers may seem counterintuitive, although classical cases (−0.5 within O_2_^−^) do exist, as mentioned before. As our approach to calculate OS^ai^ is based on wave functions and population analysis derived from that, the latter will inevitably lead to lower occupations if ΔEN becomes tiny, even though the weighting factor leads to heterolytic bond splitting. In contrast, classical oxidation numbers yield I^−I^ in HI *by definition*, since nobody cares that ΔEN is just 0.059, an insignificant number.

In the literature, atomic charges are sometimes rather naively used interchangeably with oxidation numbers, so in Fig. 7(b) we present, for reasons of comparison, the respective Löwdin charges automatically available^32^ in LOBSTER for the same molecules. Naturally, species with small electronegativity differences exhibit small charges and *vice versa*. The qualitative course, however, is far less convincing for the Löwdin charges since heterolytic bond splitting is not being performed.

For example, CsF features an almost equal charge (0.98) and oxidation score (1.00). The more covalent LiI has an oxidation score of ±0.98 and a charge of only 0.8—this is clearly a feature of the sharp assignment of bonding electron density by the weighting factor *w*_µν_ in eqn (7). For reference, we also include a correlation analogous to Pauling's in Fig. 7(c). The overall trend of experimental ionicity mirrors the course of Löwdin charges despite the former being measured spectroscopically and the latter being calculated from quantum mechanics.

## Results and discussion

### Elements

In the following section, we will illustrate the here presented method following along the classical set of oxidation-number or -state rules mentioned earlier. To do so, we have compiled a set of examples to demonstrate how the method works in the context of any chemist's intellectual training. Our analysis begins with the first rule which states that *the oxidation number of an atom in the elementary state is zero*. Fig. 8 depicts three elements all showing the expected (trivial) *ab initio* oxidation score (OS^ai^) of ±0. For comparison, we have also included Löwdin charges that arrive at the likewise trivial result—no electron transfer.

**Fig. 8:**
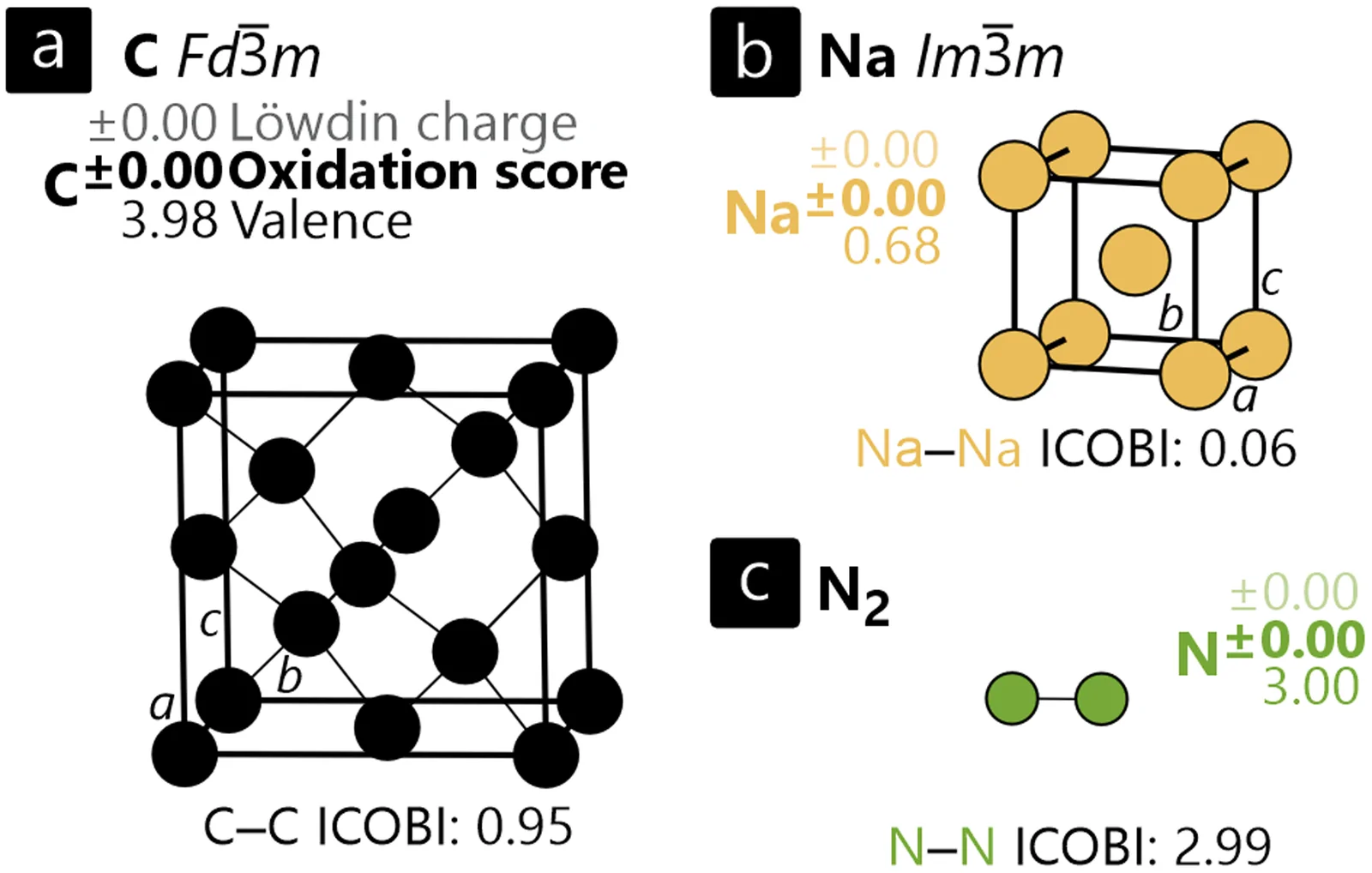
Löwdin charges, *ab initio* oxidation scores, ICOBI values (=bond orders) and valences for elemental (a) diamond-like carbon, (b) body centered-cubic sodium and (c) molecular N_2_.

In the case of carbon^33^ as shown in Fig. 8(a) the valence (=3.98) is rather close to the expected four bonds (tetravalency) a carbon atom typically forms. In elemental sodium^34^ (given in b), the valence of 0.68 may not be intuitive straight away but easily explained by the nearest-neighbour Na–Na ICOBI. As defined previously, an atom's valence is the sum of all bonds it forms to all its neighbours. Upon summing up the ICOBI values for the eight nearest-neighbour bonds (8 × 0.06 = 0.48), the missing 0.68–0.48 = 0.20 is due the six second-nearest Na–Na bonds, each contributing 0.033 to the total valence. This is in harmony with very early geometrical findings for the “effective” coordination number in bcc structures, 9.6 instead of 12.^35^ Almost needless to say, the method is not limited to solid matter, so we are showing results for the isolated dinitrogen N_2_ molecule in Fig. 8(c). Note how the valence, given in bold letters, arrives at the expected value of 3.00 indicating a triple bond.

### Alkaline metals in compounds

As homolytic partitioning of homonuclear bonds in elements works trivially, we move on to systems where heterolytic charge allocation is a must. For now, we stick to simple rock-salt-like crystal structures and discuss the fourth rule which states that *alkaline metals in compounds have the oxidation state +I while alkaline earth metals adopt the oxidation state +II*. Similar to the preceding molecular diatomics in Fig. 7(a), the rock-salt OS^ai^ are in agreement with the trends derived from ΔEN.

Fig. 9 corroborates the fourth rule for the solid-state alkali halides. Here, the OS^ai^ were calculated for the rock-salt polymorphs of the alkali metals Li, Na, K and Cs combined with the halogens F, Cl, Br, and I, and then plotted against the difference in Allen electronegativity. We note that all OS^ai^ are reasonably close to the classical ON of +I and increase, as expected, with the difference in EN. Non-integer OS^ai^, in particular seen for the least ionic LiI, are a quantum-chemical consequence, different from oxidation numbers in which iodine is counted as −I, irrespective of whatever the electronic situation. An alkaline-earth metal will be covered in the next subsection.

**Fig. 9:**
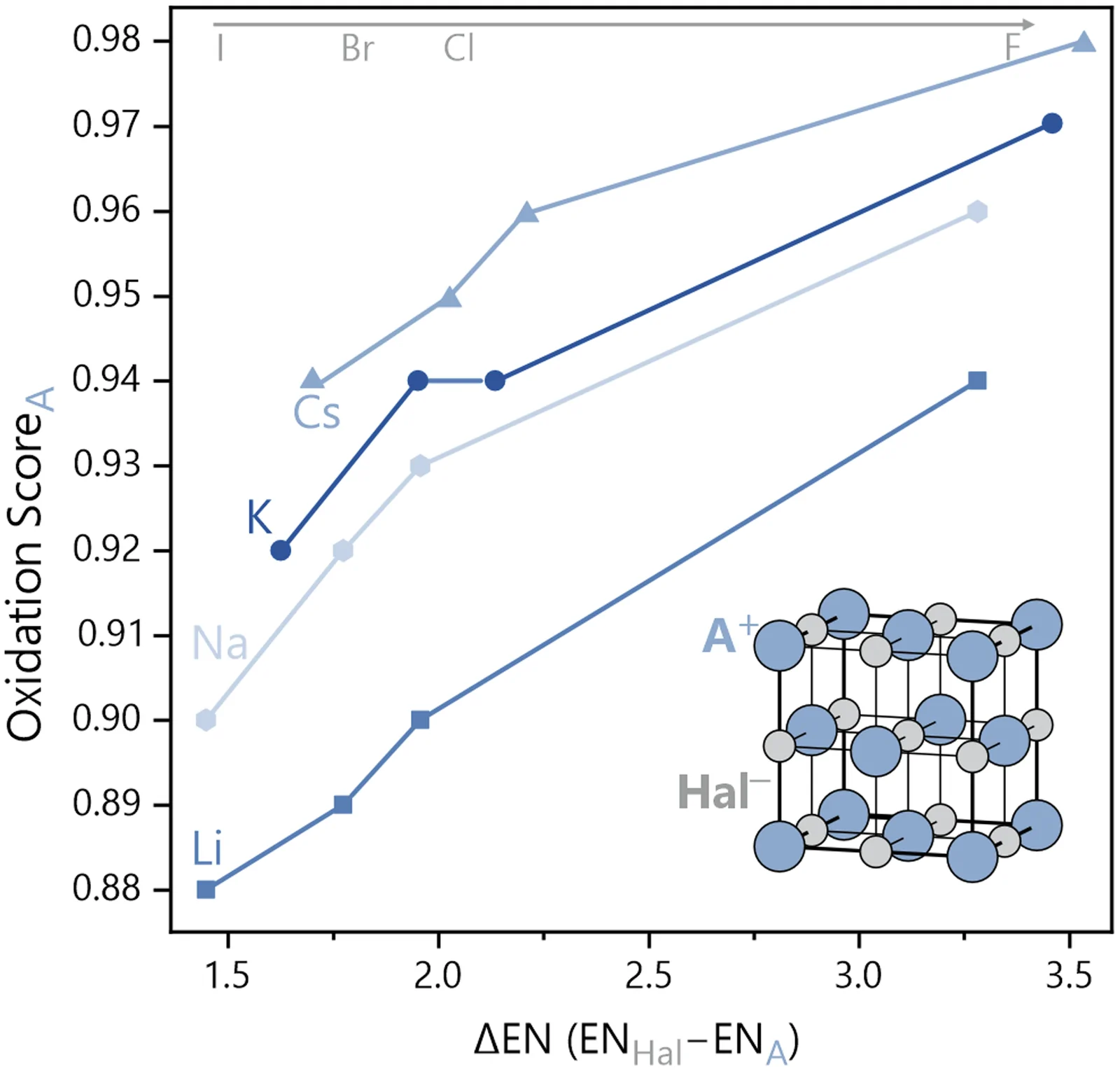
*Ab initio* oxidation scores and differences in Allen electro-negativity for the alkali halides.

### Hydrogen in compounds

We now jump to the fifth rule and also cover the second and third rules on the fly. According to the fifth rule *the oxidation number of hydrogen in compounds is +I with the exception of hydrides where it is −I*. Fig. 10 shows a collection of different hydrogen-containing compounds. The crystal structure of carbonic acid^36^ in Fig. 10(a) was selected as an example for the first part of the fifth rule, and the OS^ai^ for H arrives at +0.79, much closer to the fictitious value of +I than the Löwdin charge with a value of only +0.35. This difference lies in the homolytic transfer of bonding electrons in Löwdin's approach, as discussed above.

**Fig. 10:**
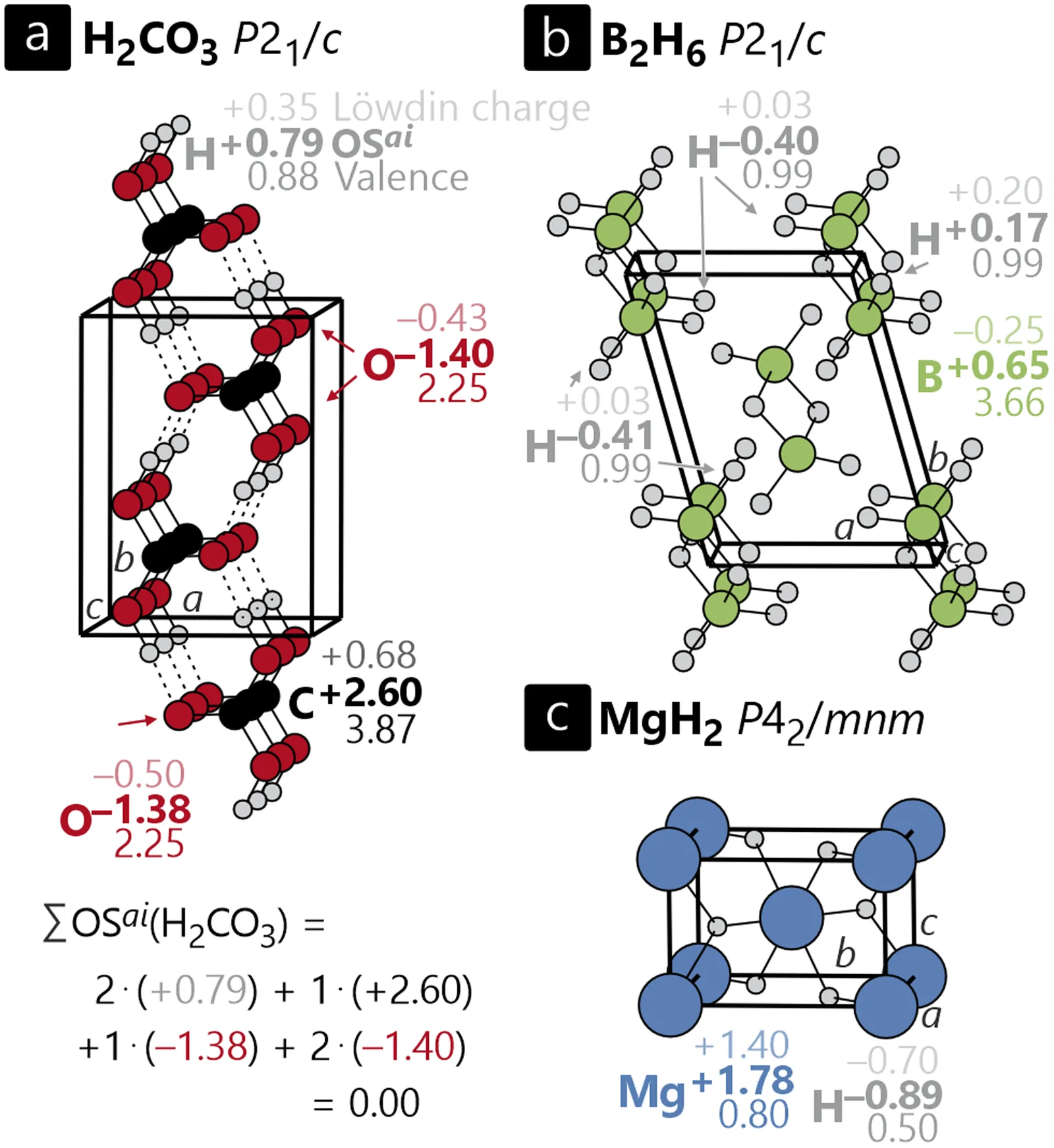
Löwdin charges, *ab initio* oxidation scores and valences of different hydrogen-containing compounds. (a) Carbonic acid serves as example for the intuitively expected ON of H. The summation shows that the OS^ai^ add up to zero. (b) The diborane example demonstrates that the method works for systems with small ΔEN. The hydride MgH_2_ in (c) is included as another exception to the classical ON of +I for H, but confirmation of the fourth rule (+II for alkaline-earth metals).

The third rule states that the algebraic sum of the oxidation numbers of the atoms in a compound is zero. Fig. 10(a) verifies this requirement from the summation of the OS^ai^ for carbonic acid, arriving at zero. Note that the carboxyl and hydroxyl O atoms do not differ significantly, about −1.40, so carbon arrives at +2.60. Due to the large covalency involved, even a higher *α* parameter will not allow the bond splitting to become “more heterolytic”, so there are not enough electrons to redistribute to arrive at −2.0 and +4.0, simply put. The classical oxidation number of +IV for carbon is a consequence of oxygen's oxidation number *defined* as −II.

The diborane^37^ molecule as part of a crystal in Fig. 10(b) serves as a good example for an exception to the ON of +I, and it also shows that even for small ΔEN the second rule is fulfilled: in a compound, the oxidation number of the element with the lower electronegativity is positive and that of the element with the higher electronegativity is negative. The Allen electronegativities Fig. 1 are 2.3 for H and 2.051 for B, making H the slightly more electronegative element, so we would expect classical oxidation numbers of −I for hydrogen and +III for boron.

Quantum chemistry yields OS^ai^ values being indeed negative for the terminal H atoms (−0.40) and positive for the B atoms (+0.65), in addition to a slightly positive value for the bridging H (+0.17). To put these numbers into perspective, in particular by comparing them with classical oxidation numbers, we remind the reader that the concept of electronegativity refers to an atomic quantity summarizing over all atomic energy levels (orbitals) and therefore loses justification when it comes to individual levels. As such, it can be reasonable to compare values referring to orbitals such as ionization energies of historical importance within semi-empirical extended Hückel theory.^38^ Given the values for the respective valence orbitals in the diborane molecule, the energetic order of 2s_B_ < 1s_H_ < 2p_B_ is present for both our *ab initio* approach and the ionization energies from the literature.^39^ We can therefore conclude that a clear shift of density from boron to hydrogen does not reflect the given orbital structure which, instead, shifts electrons in two opposite directions: the density arising from the 2s_B_–1s_H_ bond goes to boron, while the density from the 2p_B_–1s_H_ bond is shifted towards hydrogen. Alternatively expressed, the classical picture (−I for H and +III for B) is theoretically inappropriate, despite being depicted in standard textbooks, just like the Löwdin charges in Fig. 10(b), positive for H and negative for B.

A far simpler hydride case is given by MgH_2_ (ref. 40) as presented in Fig. 10(c) for which H is expected to adopt an ON of −I. Here, both OS^ai^ and Löwdin charges arrive at the anticipated negative values while the OS^ai^ are even closer to the ideal value of −I for H and +II for Mg. This paints an ionic picture in line with the small valences of 0.50 for H and 0.80 for Mg.

### Oxygen in compounds

The sixth rule deals with oxygen-containing compounds and states that *the ON of O is −II with the exception of peroxides where it is −I and fluorides where it is +II*. A common solid-state textbook example for the oxygen ON is the group of manganese oxides. Naturally, the OS^ai^ were also field tested for these systems (three of which require spin polarization; magnetic materials), and the results are given in Fig. 11. Starting off with MnO,^41^ an ON of +II and −II are expected for Mn and O, respectively, upon heterolytical charge allocation, as shown in Fig. 11(a). With values of ±1.81, the OS^ai^ actually come quite close to the anticipated integer ON and are even closer to them than the Löwdin charges with values of ±1.27. MnO is seemingly a simple case.

**Fig. 11:**
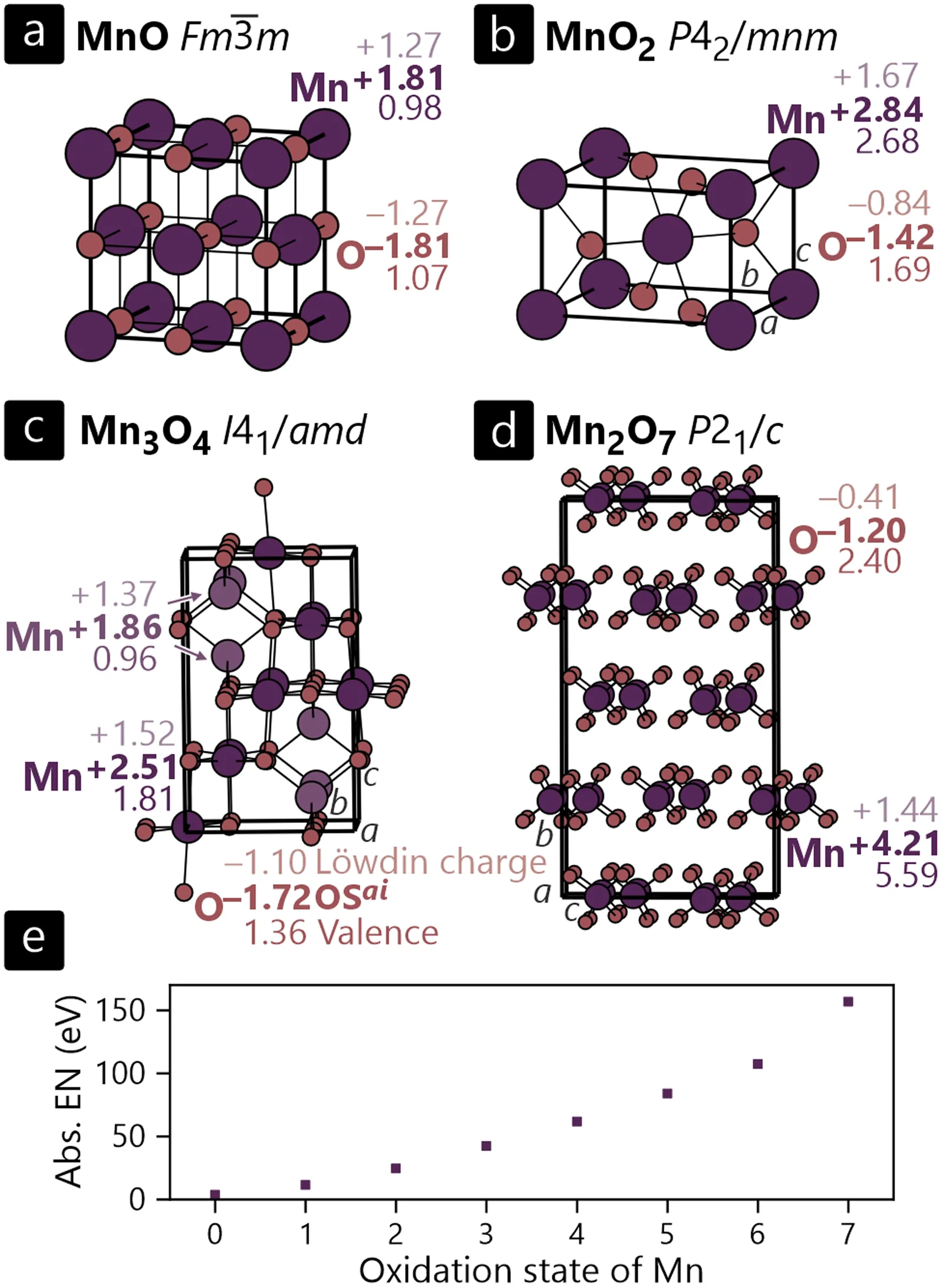
(a–d) Löwdin charges, *ab initio* oxidation scores and valences for different manganese oxides. (e) Absolute electronegativity of neutral as well as charged manganese up to Mn^7+^.

The calculated OS^ai^ for MnO_2_ (ref. 42) in Fig. 11(b) may seem a bit more puzzling, at first sight. The fifth rule is equivalent to a manganese ON of +IV but the OS^ai^ arrive at +2.84, with the Löwdin charge being even smaller. Just like for MnO, the oxidation number is not reached, the reason being that even more covalency is involved, so a decisively ionic notion is no longer valid, even though an oxidation number of −II for O would require just that. Likewise interesting is the case of Mn_3_O_4_ (ref. 43) since Mn is anticipated to exist in two distinct oxidation numbers to balance out the four O anions, then yielding a formal (Mn^+III^)_2_Mn^+II^(O^−II^)_4_ picture. A glance at Fig. 11(c) shows that less covalency is at play compared to MnO_2_ but more than in MnO, so the ionic picture is almost valid. Second, the OS^ai^ allow for the identification of two different Mn oxidation numbers also coming close to the empirical ON. That is to say that the anticipated (Mn^+III^)_2_Mn^+II^(O^−II^)_4_ is mirrored as (Mn^+2.51^)_2_Mn^+1.86^(O^−1.72^)_4_ based on OS^ai^, in rather good agreement with the ionic picture.

In the series of binary manganese oxides, the ultimate test for higher oxidation numbers is Mn_2_O_7_,^44^ the archetype of +VII for an ON. One glance at the OS^ai^, as shown in Fig. 11(d), reveals that Mn indeed acquires a rather large OS^ai^, namely +4.21, but it is still far lower than the expected classical ON. Mirroring that finding, the OS^ai^ of O (−1.20) is also much lower than the anticipated −II. At the same time, the extremely high valence of Mn (5.59)—more than twice the valence for MnO_2_—indicates an extraordinarily strong covalent character of the Mn–O bonding in Mn_2_O_7_, not too unexpectedly, since highly charged cations polarize the surrounding anions, and hence covalency may set in. Intuitively, the reason for the “too small” OS^ai^ of both Mn and O is rather clear: just like in carbonic acid, the amount of covalency is too large to allow for an ionistic picture, irrespective of the *α* parameter used. The same problem does not exist for the classical approach because as long as oxygen is *defined* as being −II, manganese can only arrive at +VII as the stoichiometry enforces that.

Since we just introduced the valence here as a handy new descriptor for the overall covalent bond capacity, it is straightforward to also numerically and visually demonstrate the increase in covalency going from MnO *via* MnO_2_ to Mn_2_O_7_ using a better-known descriptor, *i.e.*, the crystal orbital bond index (COBI) as depicted in Fig. 12.

**Fig. 12:**
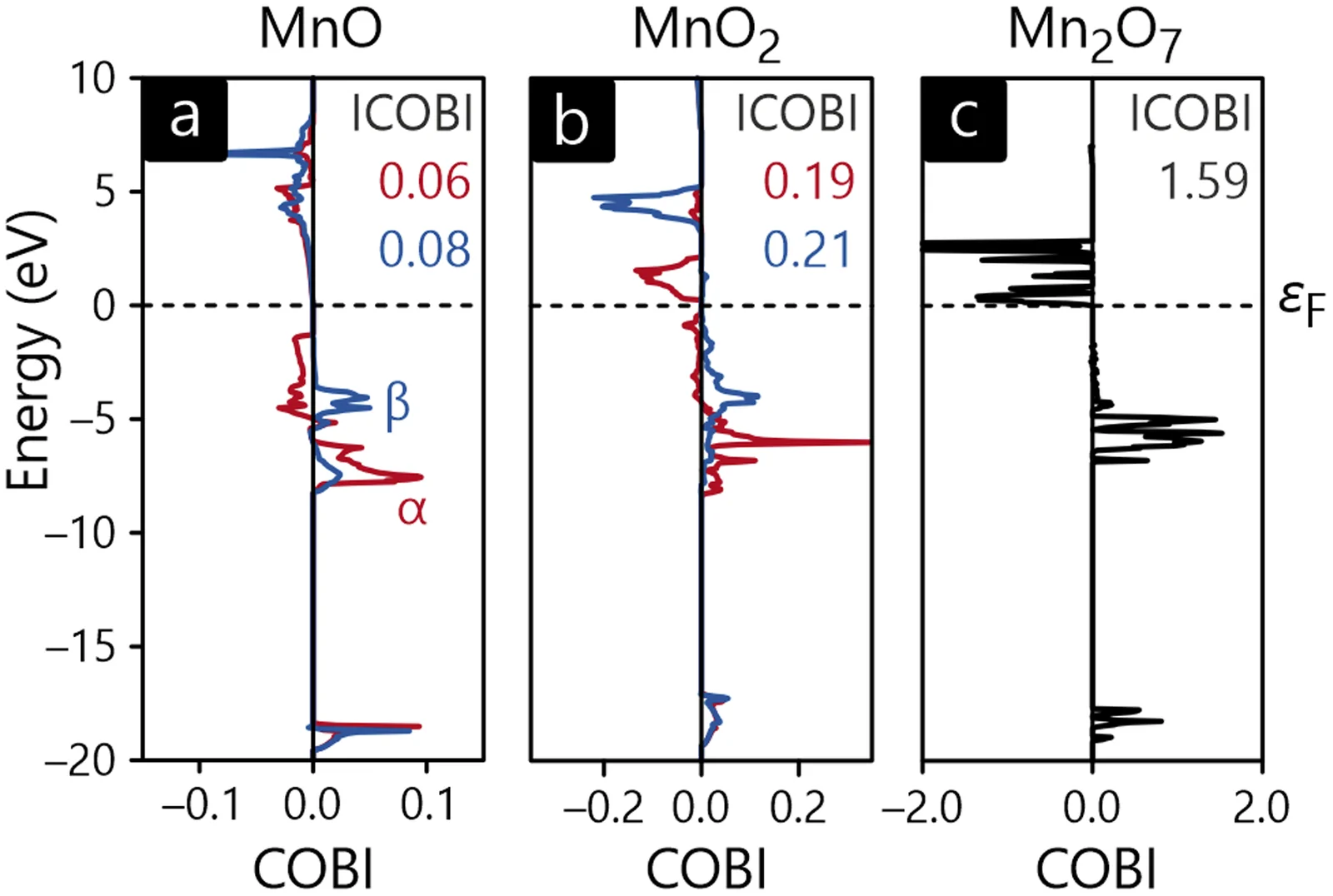
Spin-polarized (a and b) and non-spin-polarized (c) Crystal Orbital Bond Index (COBI) and its integrated value for the strongest Mn–O bond in MnO, MnO_2_ and Mn_2_O_7_, respectively, with majority/minority spins in red/blue.

As expected, the COBI and its integrated values (ICOBI) for the strongest Mn–O bond increase steadily upon going from MnO to MnO_2_ and finally to Mn_2_O_7_, respectively. In the ionic MnO, the ICOBI values are the smallest, and there are even anti-bonding contributions below the Fermi level, hardly so for MnO_2_ and none whatsoever for Mn_2_O_7_. Hence, increasing ICOBI values in that course confirm the increase in covalency. We also note that in both magnetic calculations the bonding in the α majority spin channel (in red) comes out smaller than in the β minority channel (in blue), a well-known consequence of the exchange hole^45^ and its associated contracted/expanded α/β spin orbitals.

For an in-between summary of the course of the manganese OS^ai^, two observations are relevant: first, the *ab initio* oxidation score increases with increasing empirical oxidation numbers, matching our expectations. Second, the difference between both numbers gets larger for manganese in higher oxidation numbers, simply because the anionic O is defined as −II, irrespective of its electronic situation, ionically or covalently bonded. An alternative, yet equivalent view results from considering the electronegativities of manganese in different oxidation numbers. Pauling's definition of electronegativity and Allen's implementation refer to neutral atoms which may look tolerable for Mn^+II^ but certainly not for Mn^+VII^. Mulliken's definition,^46^ while, on the other hand, coinciding with the absolute electronegativity resulting from ionization potential and electron affinity, can be derived spectroscopically for atoms as well as ions.^47^ The absolute electronegativities measured in eV are given in Fig. 11(e) for Mn^0^ up to Mn^+VII^. To no surprise, the electronegativity of neutral manganese is the smallest, and it heftily increases with the oxidation number. The maximum value of 156.9 eV is reached for Mn^+VII^, almost two orders of magnitude larger than that for Mn^0^, mirroring the enormous tendency to attract electrons for such a large positive charge. That being said, it appears that +VII for Mn is unlikely to exist given an oxygen surrounding, even though it is defined as such by the stoichiometry.

Our analysis of oxygen-containing compounds would be incomplete without taking a look at further textbook examples. The sixth rule states that peroxides are a special case where O adopts an ON of −I. The crystal structure of hydrogen peroxide^48^ is given in Fig. 13(a) along with the Löwdin charges, OS^ai^ and valences. Clearly, the OS^ai^ match the expected integer values of ±I better than the Löwdin charges, and ±0.79 for the OS^ai^ also fulfill the expectations of a peroxide O, unlike −II. An analysis of the valences shows that O forms the expected two bonds (valence of 2.06), and hydrogen forms slightly less than one bond (valence of 0.88).

**Fig. 13:**
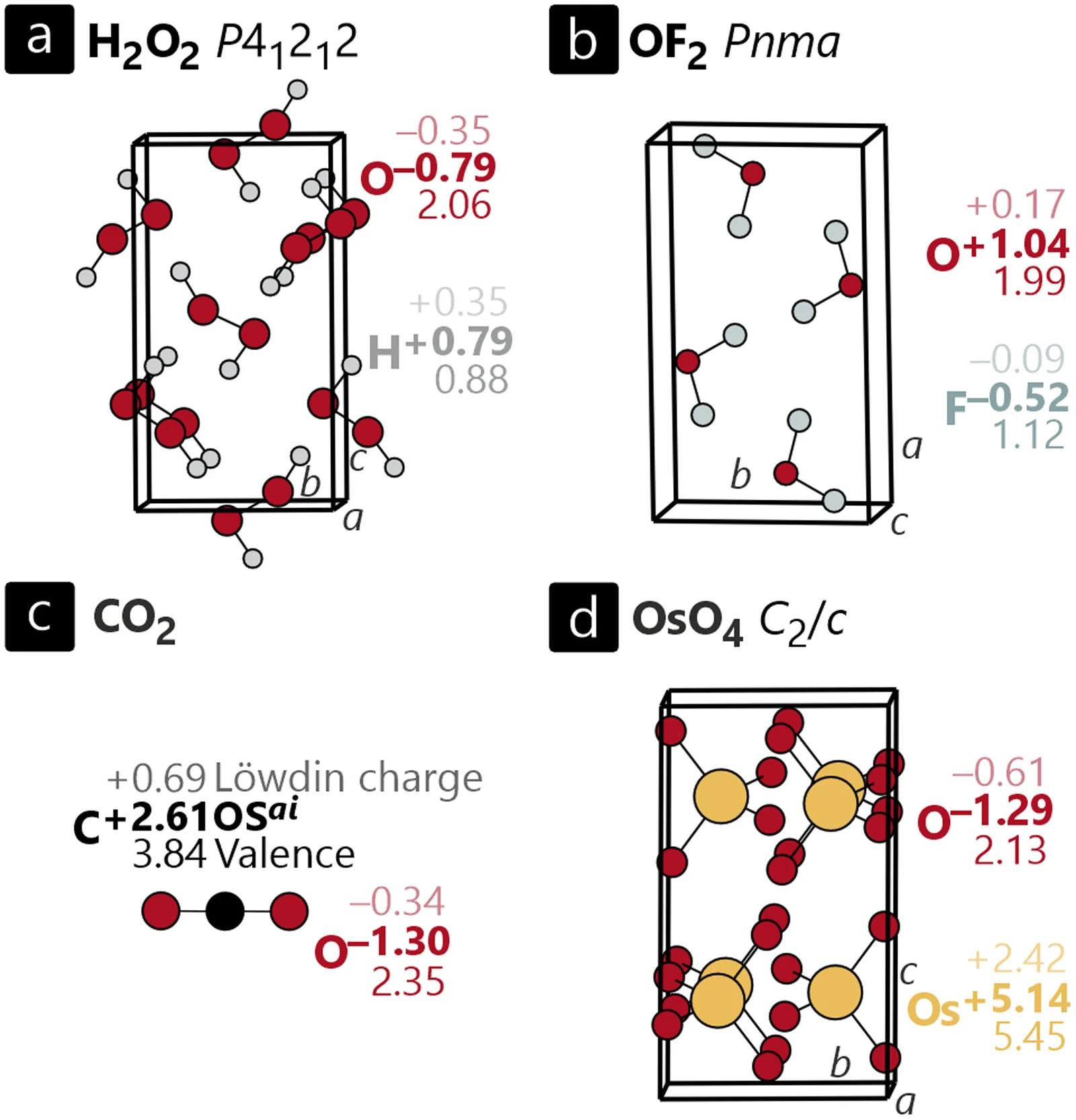
(a–d) Löwdin charges, *ab initio* oxidation scores and valences for textbook examples of oxides.

Another compound to be discussed in the context of the sixth rule is oxygen difluoride shown in Fig. 13(b) by its crystal structure.^49^ It is a special case since O is expected to be cationic, adopting an ON of +II due to its smaller EN (3.610 for O and 4.193 for F, see Fig. 1. In terms of the OS^ai^, the molecular system is formulated as O^+1.04^(F^−0.52^)_2_. While this result shows that positive and negative charges are assigned correctly to the respective atoms, the charge transfer, however, is smaller than expected, a consequence of the energetic proximity of O 2p and F 2p, so there is no large difference in their gross orbital population, and ICOBI = 0.98 indicates substantial covalency.

Likewise, there is molecular CO_2_, shown in Fig. 13(c). Here the OS^ai^ are somewhat smaller than anticipated upon heterolytical charge allocation, and the molecule is far from being ionic, not too surprisingly. Mirroring this, the valences corroborate the expected strong covalency with carbon almost forming four bonds (3.84) and oxygen forming even more than two bonds (2.35).

To conclude the textbook examples for oxygen in compounds, we also tested the OS^ai^ on OsO_4_,^50^ and the results are depicted in Fig. 13(d). Similar to the very high Mn oxidation numbers presented before, the OS^ai^ values come out lower than in the empirical Os^+VIII^(O^−II^)_4_. Os arrives at an OS^ai^ of 5.14 and a valence of 5.54. The difference between the OS^ai^ and empirical ones is again rationalized by the strongly charged Os cation, analogous to the Mn_2_O_7_ discussed before and the high amount of covalency.

### Fluorine compounds

The last rule on our list is the seventh one which concludes that *fluorine in a compound has the oxidation state −I* as a result of its highest electronegativity compared to all other elements (except neon). Fig. 14 compiles four fluorides^51–54^ ranging from the expected ON of +II for Xe in XeF_2_, as shown in Fig. 14(a), up to +VI for Te in TeF_6_, as shown in Fig. 14(d). The OS^ai^ for F span from −0.75 to −0.86 which we consider close enough to −I to conclude that the algorithm for heterolytic charge allocation works, as expected in these rather ionic examples. In the Xe compounds, as shown in Fig. 14(a and b), the charge transfer is slightly smaller than in the tellurium compounds, in line with the smaller difference in electronegativity between Xe and F (ΔEN = 1.611) than between Te and F (ΔEN = 2.035). For large empirical oxidation numbers, OS^ai^ come out smaller, as seen before, proving significant covalency once again.

**Fig. 14:**
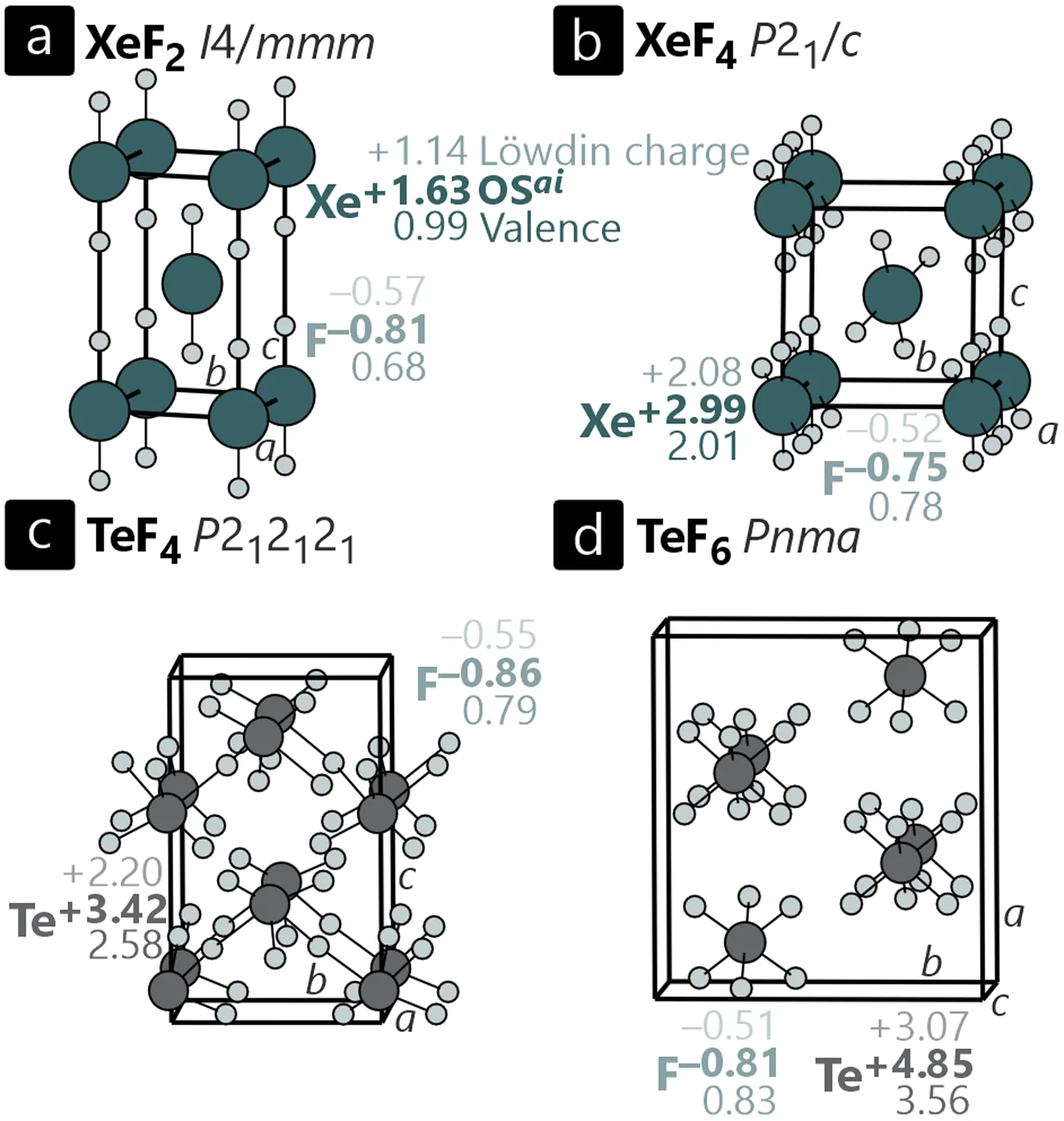
(a–d) Löwdin charges, *abinitio* oxidation scores and valences for different fluorides.

### Non-trivial cases

After having evaluated the OS^ai^ performance in the context of the classical rules for oxidation-number assignment, this section will be dedicated to systems where assigning oxidation numbers is no longer trivial or even ambiguous. For example, metallic systems are chronically difficult due to their delocalized character, so textbook rules (where electrons add up nicely) are no longer suitable to arrive at a simple oxidation-number description. We therefore aim to demonstrate whether or not the aforementioned OS^ai^ can aid in such an endeavour, the most crucial challenge. Fig. 15 and 16 depict seven characteristic examples of systems of that type.

**Fig. 15:**
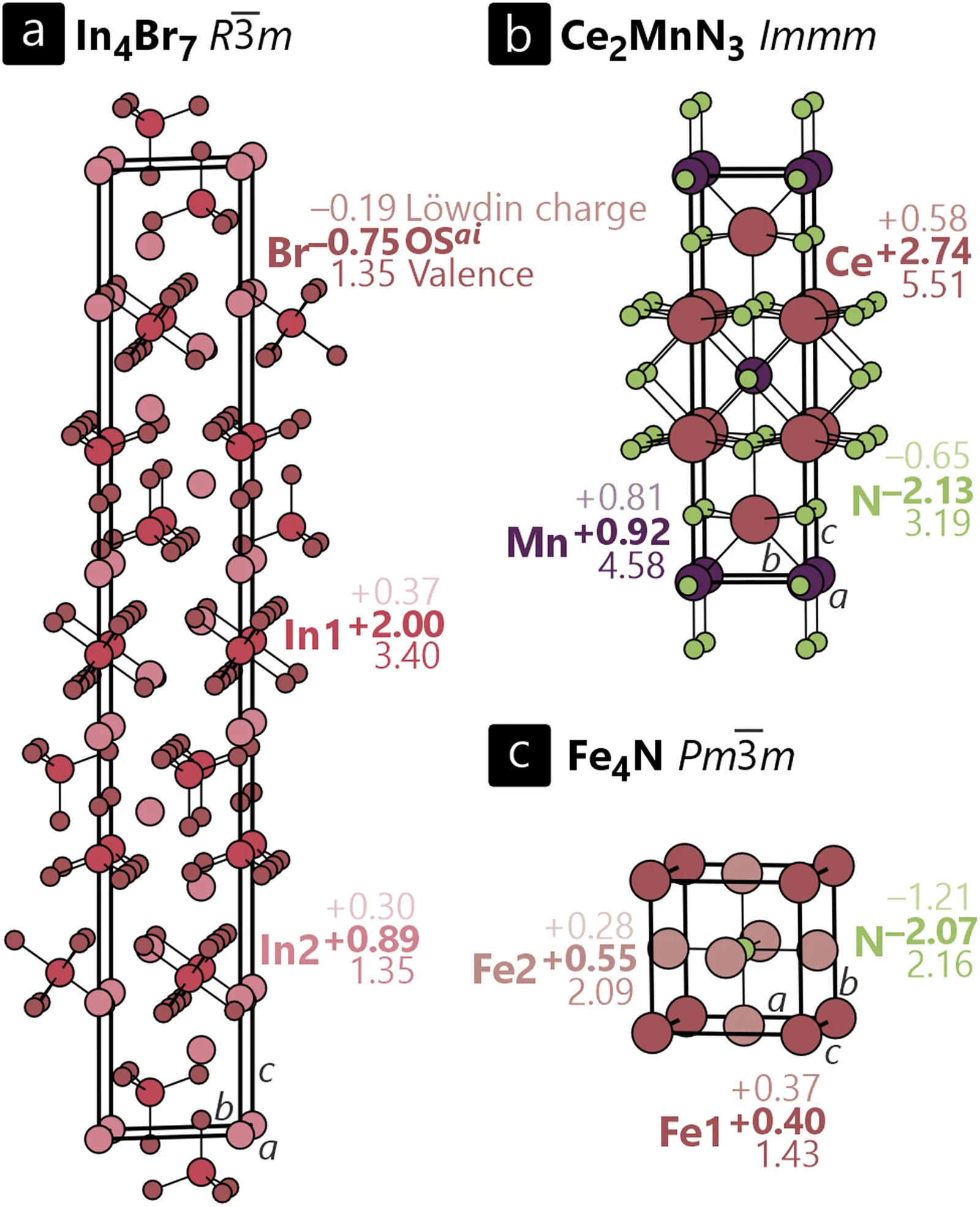
Löwdin charges, *ab initio* oxidation scores and valences for (a) In_4_Br_7_, (b) Ce_2_MnN_3_, and (c) Fe_4_N.

**Fig. 16:**
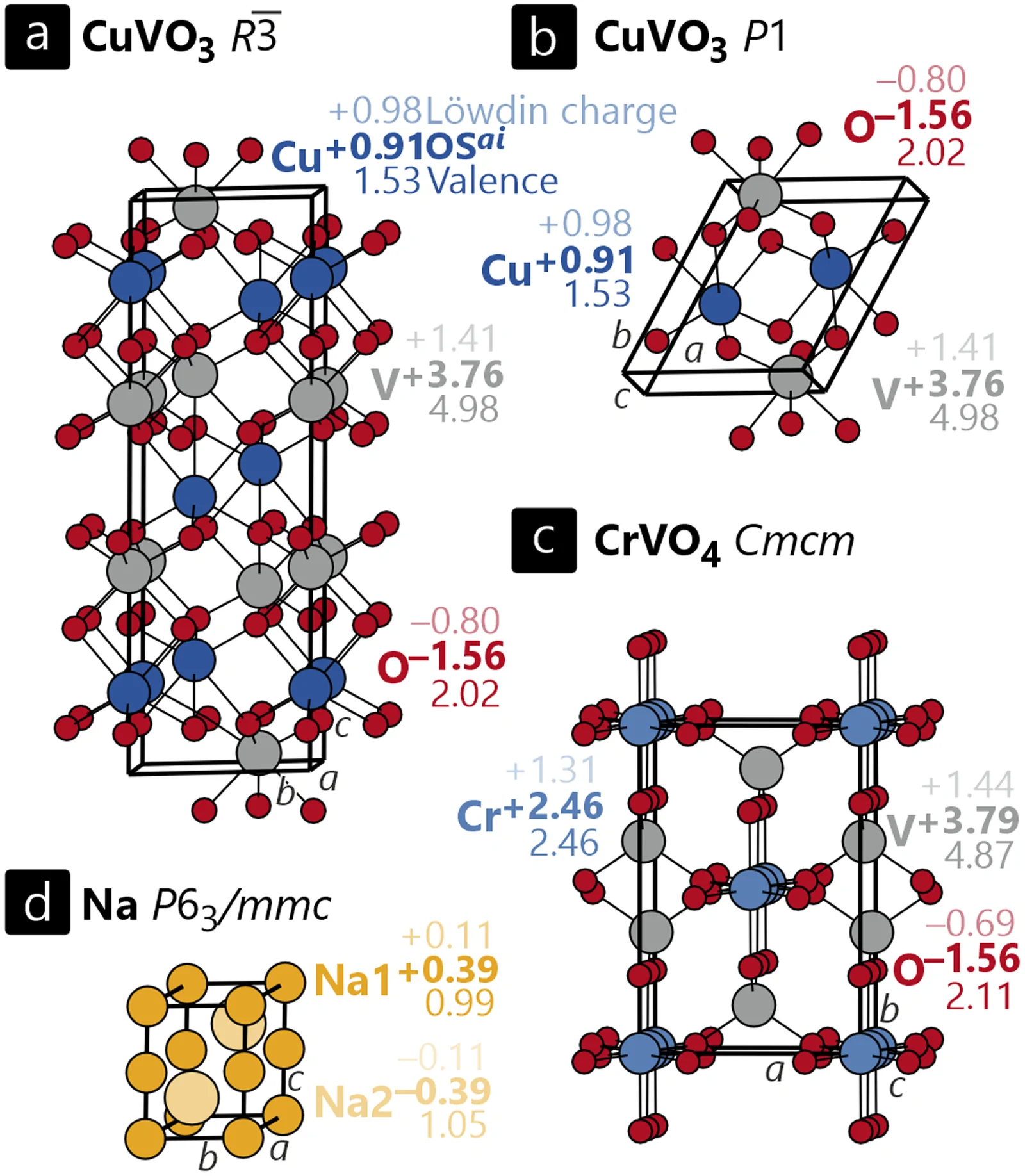
Löwdin charges, *ab initio* oxidation scores and valences for CuVO_3_ in the (a) rhombohedral *R*3̄ and (b) the triclinic *P*1 structure, in (c) for CrVO_4_, and in (d) for extreme-pressure transparent Na.

Starting off with semiconducting In_4_Br_7_, Fig. 15(a), the assignment of an ON to Br is rather trivial since the textbook rules require ON = −I for Br. As regards indium, there is an obvious discrepancy in the literature. While the original paper^55^ finds two different In species and yields (In^3+^)_3_(In^+^)_5_(Br^−^)_14_ = In_8_Br_14_ = 2 × In_4_Br_7_, in harmony with crystal chemistry, the Materials Project Database,^56^ for example, does not make such a cationic distinction. Even though the two symmetry-inequivalent (small) In^3+^ adopt tetrahedral/octahedral coordination whereas 10–12-fold coordination is found for the three symmetry-inequivalent (large) In^+^, the Materials Project arrives at a rather simple (In^+1.75^)_4_(Br^−^)_7_, incorrectly so. The OS^ai^ depicted in Fig. 15(a), however, clearly corroborate the strongly different oxidation numbers. Admittedly, the OS^ai^ do not fully reach +III and +I, but rather +2 and +0.89, mirroring significant covalency (ΔEN = 1.029). Interestingly enough, the large crystal-chemical difference mirrored by the OS^ai^ is almost hidden in the Löwdin charges, too. Although both indium species also have non-identical Löwdin charges (+0.37 and +0.30), we consider their charge difference to be too small to allow for a clear distinction, despite fundamentally different coordination polyhedra. In_4_Br_7_, by the way, is an inherently unstable (“above the convex hull”) solid which starts to decay at an In^+^ site.

Another puzzling case already mentioned in the Introduction is that of the ternary metallic nitride Ce_2_MnN_3_. Two different formulations, *i.e.*, (Ce^+III^)_2_Mn^+III^(N^−III^)_3_ and (Ce^+IV^)_2_Mn^+I^(N^−III^)_3_ were proposed and magnetic data did not allow low-spin Mn^+III^ and low-spin Mn^+I^ to be distinguished. The problem was resolved by electron-density/COHP analysis, yielding +IV for Ce and +I for Mn.^14^ In Fig. 15(b) the OS^ai^ allow for a clear distinction between Mn^+III^ and Mn^+I^ as OS^ai^ = +0.92 is much closer to +I than to +III, allowing us to comfortably confirm the Mn^+I^ notion. Admittedly, Ce does not reach the anticipated +IV (OS^ai^ = +2.74) as covalency is at play, once again, in particular in a metallic system.

We continue with the metallic nitride Fe_4_N (ref. 57) for which an ON of −III for N would be expected, then running into a problem for iron, however. Notions such as Fe^0^(Fe^+I^)_3_N^−III^ and (Fe^+0.75^)_4_N^−III^ may come to mind but a definite claim is empirically impossible. Using the OS^ai^, N arrives at −2.07, still far away from −III but certainly more reasonable than the Löwdin charge (−1.21). The unit cell contains two distinct Fe positions, with Fe1 arriving at OS^ai^ = +0.40 and Fe2 at OS^ai^ = +0.55, so this appears as rather fitting as Fe2 coordinates the anionic N whereas Fe1 is the isolated corner atom. Because the *ab initio* picture reading Fe^+0.40^(Fe^+0.55^)_3_N^−2.07^ is closer to (Fe^+0.75^)_4_N^−III^ than to Fe^0^(Fe^+I^)_3_N^−III^, we regard (Fe^+0.75^)_4_N^−III^ as the better choice.

We conclude this section by investigating three ternary oxides and one extreme-pressure phase. The oxides are CuVO_3_ in its rhombohedral^58^ and triclinic form^59^ as well as CrVO_4_,^60^ all presented in Fig. 16. In the case of CuVO_3_ shown in Fig. 16(a), the authors propose two different formulations, either as Cu^+I^V^+V^O_3_ or Cu^+II^V^+IV^O_3_, based on interatomic distances. A study based on photoemission spectroscopy states that the formulation in terms of the oxidation number is not unambiguously definable.^61^ The OS^ai^, however, point more clearly into one direction, namely that of the first formulation, so Cu^+I^V^+V^O_3_, as we receive identical OS^ai^ as well as Löwdin charges for both CuVO_3_ modifications. Since Cu acquires an OS^ai^ of +0.91, one may safely formulate it as Cu^+I^. With O not quite arriving at −II (but rather at −1.56) and the large valence of vanadium (4.98)—so there is a large amount of covalency in this system, once again—it does not come as a surprise that the OS^ai^ will not yield the ON of +V for vanadium. The notion that this V^+3.76^ corresponds to the formal V^+V^ is further corroborated by looking at CrVO_4_ given in Fig. 16(c). With a value of +3.79 the OS^ai^ of V is almost identical to the one in CuVO_3_. For CrVO_4_, there are two possible formulations to balance out the four O atoms, to be written as Cr^+III^V^+V^O_4_ and Cr^+VI^V^+II^O_4_. Quite clearly, the OS^ai^ unambiguously favour the first formulation, again featuring V^+V^. The latter notion seems rather unlikely since V would be over-shooting the formal +II and the OS^ai^ of Cr (+2.46) is certainly closer to +III than to +VI.

Eventually, there is transparent sodium, depicted in Fig. 16(d). The phase forms from metallic sodium at extreme pressures of about 200 GPa adopting the [NiAs] structure type.^62^ The presence of this very crystal structure together with the semiconducting transport property alludes to a more ionic notion in which a larger “anionic” Na builds a hexagonal close-packed (hcp) lattice in which a smaller “cationic” Na occupies the octahedral holes, even though the Löwdin charges are only ±0.11.^63^ The OS^ai^, however, corroborate the Na^+I^, Na^−I^ notion much better with values of ±0.39. So transparent sodium is an electronically binary phase, and a salt one might conveniently simplify as “Na^+^Na^−^”.

## Conclusion

Having examined both OS^ai^ and valences on a number of molecular and periodic matter, either straightforwardly oxidation number-wise or for non-trivial compounds, it is time to have a look from a distance and identify general trends. Since oxidation numbers are empirically drawn in almost any solid-state context, it is only natural to compare both methods, and the correlation between computation-based OS^ai^ and classical ON is shown in Fig. 17(a) for the textbook examples presented in this manuscript. In fact, there is an almost linear relationship between the OS^ai^ and ON, providing an a *posteriori* support of the latter (or former). In contrast to other population analyses, such as Mulliken or Löwdin charges, the OS^ai^ strongly correlate with electronegativities. This makes OS^ai^ a powerful alternative whenever charges of different elements are set into context. On average, the new OS^ai^ are smaller by a factor of about 0.70 throughout all types of compounds, from simple halides to complex magnetic oxides. This relation underlines the robustness of the method and the application to the ambiguous examples that cannot easily be solved on paper, *e.g.*, Ce_2_MnN_3_ and the CuVO_3_ phases.

**Fig. 17:**
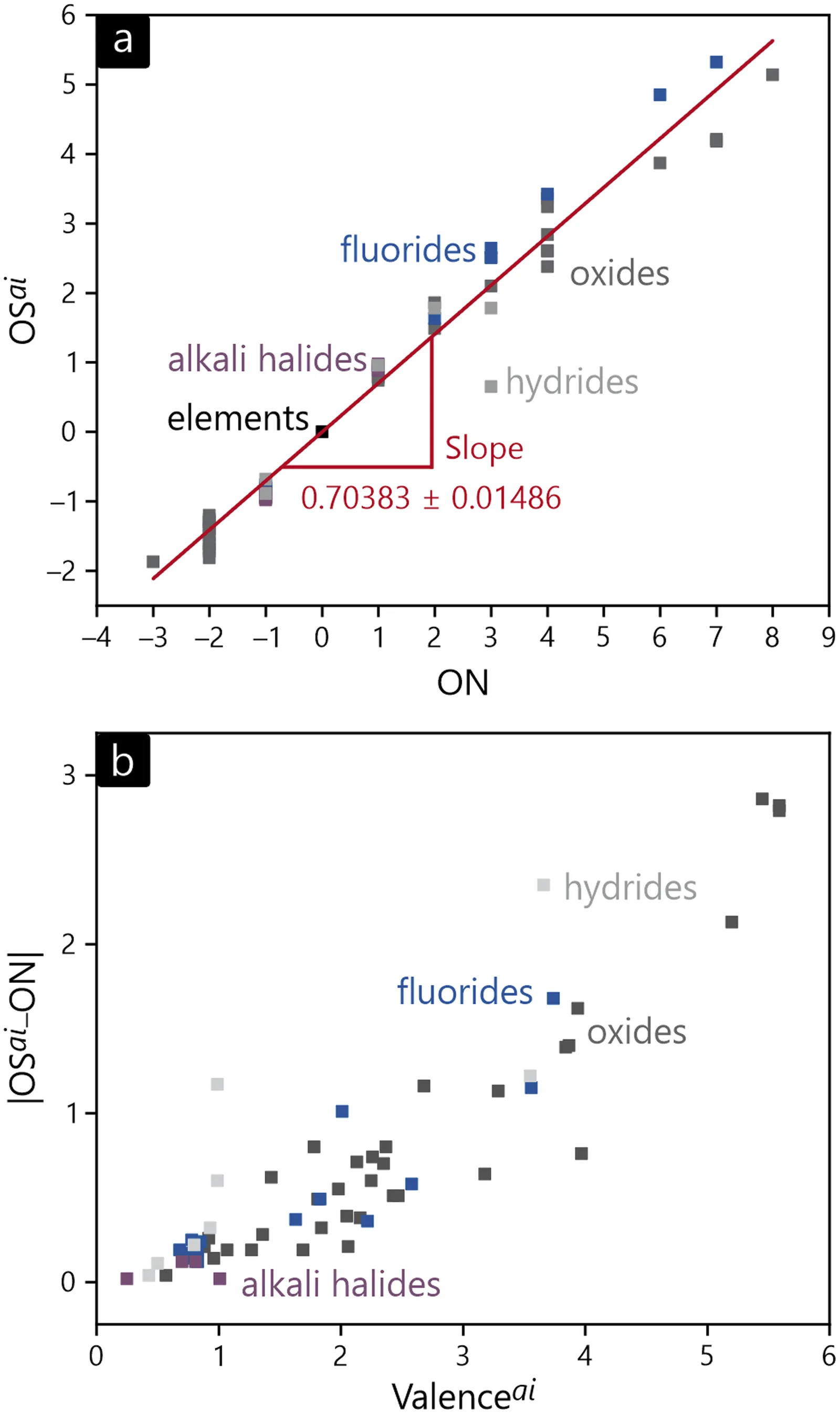
(a) Plot of computation-based OS^ai^ against classical ON showing an almost linear relationship. (b) Plot of the difference between OS^ai^ and the classical ON as a function of the computation-based valence.

Despite the linear correlation between OS^ai^ and ON, the question remains why the difference between both methods arises in the first place. To illustrate this, Fig. 17(b) presents the numerical difference between computation-based OS^ai^ and ON as a function of the atom's valence. The almost quadratic correlation highlights the importance of covalency. The plot therefore not only manifests the role played by covalency, it also suggests that the OS^ai^ mirror the bonding situation, in particular when combined with valence information; at the same time, the essential idea behind classical oxidation number assignment is retained. In case a quick renormalization of OS^ai^ to classical oxidation numbers ON were needed, however, the simple OS^ai^_renorm_ = OS^ai^/0.7 formula corresponding to Fig. 17(a) would do.

In the course of this article, we have proposed a quantum-chemical scheme to calculate *ab initio* oxidation scores OS^ai^ and valences from first principles for whatever given molecule or material. The method is able to reproduce seven well-known rules for empirical oxidation numbers, at least semi-quantitatively, for a plethora of examples. Furthermore, it aids in finding simple descriptions for non-trivial cases like metallic or other related systems where traditional oxidation number assignment is not always unambiguous. Nonetheless, deviations exist for large “textbook” oxidation numbers (say, in oxygen-rich molecular oxides), and these result from significant covalency, disagreeing with the highly simplified (ionic) textbook picture as the underlying basis of traditional oxidation numbers. We do not at all intend to question the concept of classical oxidation number assignment; quite to the contrary, we wish to point out that the new concept of OS^ai^ directly mirrors the given electronic and crystal structure, and also the orbital populations, while assigning oxidation numbers, in particular for difficult cases such as metallic compounds.

## Computational details

All structural optimizations were carried out using the Vienna *ab initio* simulation package (VASP)^64–67^ v.6.4.1 with PAW-based^68^ pseudopotential-like electronic wave functions. Electronic exchange and correlation were described using the functional of Perdew, Burke and Ernzerhof optimized for solids (PBEsol).^69^ The convergence criteria were set to energetic differences of 10^−8^ eV for electronic and 10^−6^ eV for ionic optimizations. 700 eV was chosen as an energy cutoff of the plane-wave basis set. To also account for van-der-Waals interactions, D3 correction with Becke–Johnson damping^70,71^ was applied to all systems where appropriate. *k*-point meshes were generated according to the Monkhorst–Pack scheme.^72^ Since H_2_CO_3_ is a high-pressure phase, an external pressure of 2 GPa was applied to this system during optimization; an analogous approach (200 GPa) applies to transparent Na. Magnetic orderings were used for the manganese oxides (except for Mn_2_O_7_) and for Fe_4_N as well as CrVO_4_ according to the reference given for each of the following systems: MnO,^73^ MnO_2_,^74^ Mn_3_O_4_,^75^ Fe_4_N,^57^ and CrVO_4_.^76^*U* parameters were determined in VASP using the linear response *ansatz* of Cococcioni *et al.*^77^ and applied during the optimization process. They arrived at 8.77 eV for MnO, 7.09 eV for MnO_2_, 6.91 eV for Mn_3_O_4_, 4.57 eV for Fe_4_N and 5.74 eV for CrVO_4_.

To check for the influence of the exchange–correlation functional and the plane-wave code, various test calculations for H_2_O_2_, MnO, and Mn_2_O_7_ were carried out using the LDA, PW91, PBE, PBEsol, and MBJ functionals in combination with PAW-based VASP, Quantum ESPRESSO and Abinit codes but the results hardly differ (Tables S1 to S3).

Brillouin-zone integrations were carried out using Blöchl's tetrahedron method.^78^ A development version of the Local Orbital Basis Suite Towards Electronic-Structure Reconstruction (LOBSTER)^26,79–84^ package was used to project the PAW wave function onto the all-electron Slater-type orbital basis set pbeVASPfit2015 (ref. 82) to gain the *ab initio* oxidation scores, valences and density of valences (DOV) provided in this article. To account for the 4d orbitals on Sr and the 5p orbitals on Cd, the custom basis feature in LOBSTER was used. The employed orbitals were taken from Y and In, respectively.

In order to not only compare the OS^ai^ with Löwdin charges but also with partial charges emerging from density-based space-portioning schemes, Hirshfeld and Hirshfeld-I charges were calculated using the VASP program. The calculation of Bader charges was performed with the Critic2 program v.1.2.^85,86^ using Henkelman integration.^87–89^ The results for H_2_O_2_, MnO, and Mn_2_O_7_ and OF_2_ are given in Table S7 along with a short discussion of the results.

## Author contributions

Linda S. Reitz: conceptualization, investigation, software, formal analysis, visualization, writing – original draft, writing – review & editing, project administration. Peter C. Müller: conceptualization, investigation, formal analysis, visualization, writing – original draft, writing – review & editing. Richard Dronskowski: resources, writing – review & editing, supervision, funding acquisition.

## Conflicts of interest

There are no conflicts to declare.

## Supplementary Material

SC-017-D6SC03835B-s001

## Data Availability

The original data supporting this article have been generated (and may be reproduced) from the LOBSTER code for which an updated version will be released on http://www.cohp.de after publication of this article. Supplementary information (SI): a graphical representation of eqn (6) for a CO_2_ molecule with a short figure caption as well as OS^ai^ and Löwdin charges for H_2_O_2_, MnO, and Mn_2_O_7_ as a function of various exchange–correlation functionals and plane-wave codes (Tables S1–S3). Furthermore, the influence of a slight variation of the *U* parameter on the OS^ai^ and Löwdin charges is demonstrated in Tables S4 and S5 for MnO and MnO_2_ yielding no significant change in the respective results. Basis set sensitivity was tested (Table S6) for the LOBSTER-available basis sets Bunge and pbeVASPfit2015, also showing no difference in the respective OS^ai^. The OS^ai^ are also compared with partial charges calculated using a density-based partitioning scheme (*i.e.*, Hirshfeld, Hirshfeld-I and Bader charges). The results are given in Table S7 with a small discussion of the results. Additional information on the choice of the *α* parameter is given in Fig. S2. Ref. 90 is cited exclusively in the SI. See DOI: https://doi.org/10.1039/d6sc03835b.
