# Supplementary material for: Generalized valences and oxidation scores for solids
Source: Chem Sci. 2026 Sep 2;17(42):20431–45. doi: 10.1039/d6sc03835b (PMC13575970; doi:10.1039/d6sc03835b)
Supplement: Supplementary file 1 [file SC-017-D6SC03835B-s001.pdf]

Supplementary Information (SI) for Chemical Science.
This journal is © The Royal Society of Chemistry 2026

Generalized Valences and Oxidation Scores for Solids

Linda S. Reitz, Peter C. Müller, and Richard Dronskowski*

Chair of Solid-State and Quantum Chemistry, Institute of Inorganic Chemistry, RWTH Aachen University, 52056 Aachen, Germany.

Supplementary Information

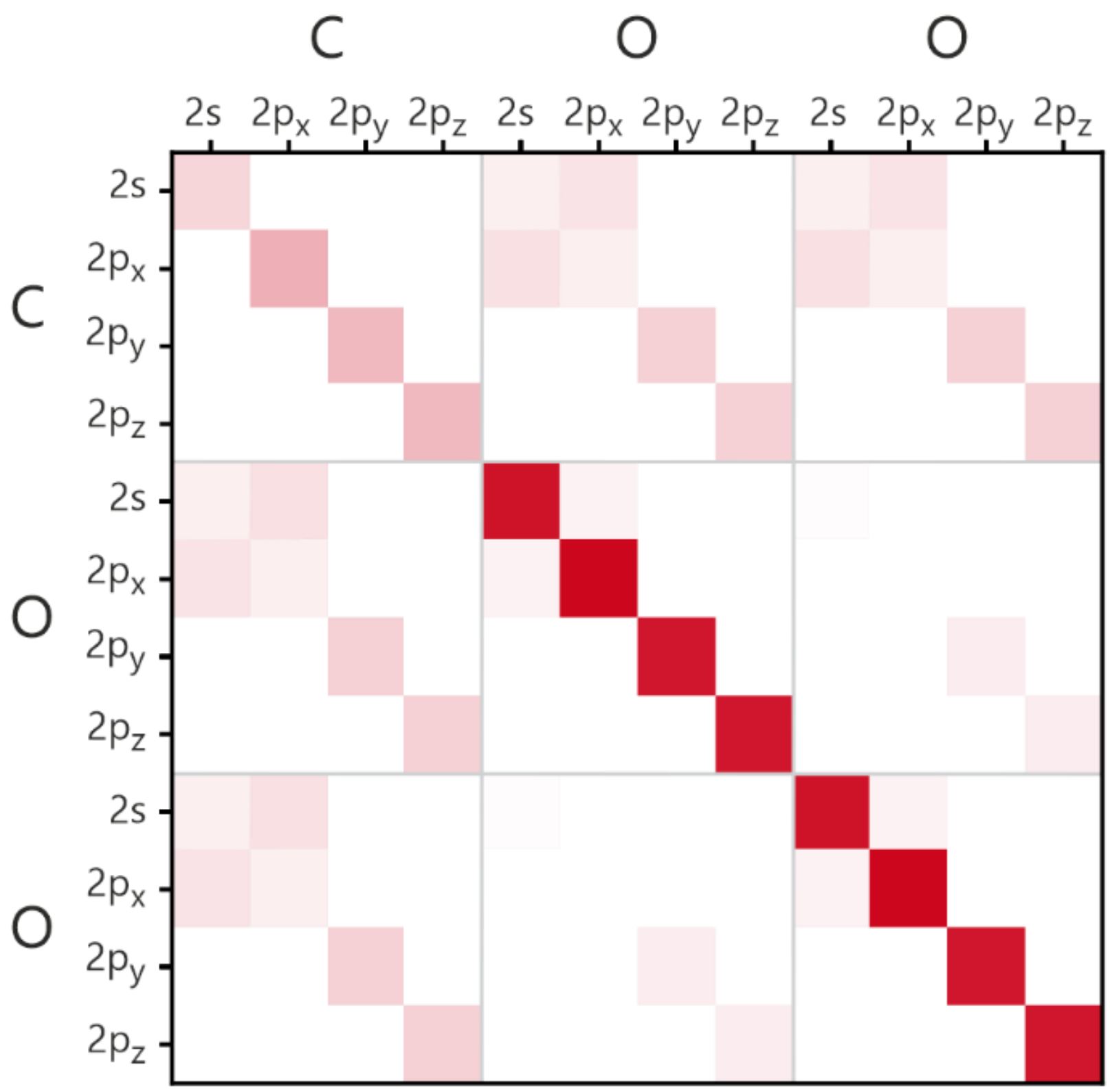

Figure S1. Heat map of the trace of the density matrix, Tr $(0.5P^2)$, broken down into AO–AO contributions. The figure exemplifies eq. 6 of the manuscript by the CO_2 molecular example. The atom-centered contributions are blocked on the matrix diagonal while the bond-centered contributions are on the off-diagonal blocks. Notably, the sum of all AO–AO pairs of the off-diagonal blocks equals the Wiberg bond index between the corresponding atoms whereas the sum along one (horizontal or vertical) direction of this matrix, i.e., over all orbitals *v*, equals the Löwdin population of this orbital. The sum of all values exactly yields the total number of 16 valence electrons.

Table S1a. OS^{ai} for Hydrogen Peroxide calculated using different methods and codes.

Atom	VASP (PAW/GGA/ PBEsol)	VASP (PAW/ LDA)	VASP (PAW/meta-GGA/MBJ)	VASP (PAW/GGA/ PW91)	QE (PAW/GGA/ PBEsol)	Abinit (PAW/GGA/ PBE)
O	−0.79	−0.79	−0.81	−0.79	−0.78	−0.78
H	+0.79	+0.79	+0.81	+0.79	+0.78	+0.78

Table S1b. Löwdin Charges for Hydrogen Peroxide calculated using different methods and codes.

Atom	VASP (PAW/GGA/ PBEsol)	VASP (PAW/ LDA)	VASP (PAW/meta-GGA/MBJ)	VASP (PAW/GGA/ PW91)	QE (PAW/GGA/ PBEsol)	Abinit (PAW/GGA/ PBE)
O	−0.35	−0.35	−0.38	−0.35	−0.35	−0.34
H	+0.35	+0.35	+0.38	+0.35	+0.35	+0.34

Table S2a. OS^{ai} for MnO calculated using different methods and codes.

Atom	VASP (PAW/GGA/ PBEsol)	VASP (PAW/ LDA)	VASP (PAW/meta-GGA/MBJ)	VASP (PAW/GGA/ PW91)	QE (PAW/GGA/ PBEsol)	Abinit (PAW/GGA/ PBE)
O	−1.81	−1.80	−1.88	−1.82	−1.78	−1.77
Mn	+1.81	+1.80	+1.88	+1.82	+1.78	+1.77

Table S2b. Löwdin Charges for MnO calculated using different methods and codes.

Atom	VASP (PAW/GGA/ PBEsol)	VASP (PAW/ LDA)	VASP (PAW/meta-GGA/MBJ)	VASP (PAW/GGA/ PW91)	QE (PAW/GGA/ PBEsol)	Abinit (PAW/GGA/ PBE)
O	−1.27	−1.26	−1.44	−1.29	−1.23	−1.13
Mn	+1.27	+1.26	+1.44	+1.29	+1.23	+1.13

Table S3a. OS[ai] for Mn_2O_7 calculated using different methods and codes.

Atom	VASP (PAW/GGA/ PBEsol)	VASP (PAW/ LDA)	VASP (PAW/meta-GGA/MBJ)	VASP (PAW/GGA/ PW91)	QE (PAW/GGA/ PBEsol)	Abinit (PAW/GGA/ PBE)
O	−1.20	−1.20	−1.24	−1.20	−1.24	−1.21
Mn	+4.21	+4.19	+4.36	+4.21	+4.37	+4.24

Table S3b. Löwdin Charges for Mn_2O_7 calculated using different methods and codes.

Atom	VASP (PAW/GGA/ PBEsol)	VASP (PAW/ LDA)	VASP (PAW/meta-GGA/MBJ)	VASP (PAW/GGA/ PW91)	QE (PAW/GGA/ PBEsol)	Abinit (PAW/GGA/ PBE)
O	−0.41	−0.41	−0.47	−0.41	−0.55	−0.42
Mn	+1.44	+1.42	+1.62	+1.45	+1.93	+1.48

Table S4a. OS[ai] for MnO calculated with *U* parameters ranging from 6 to 10 eV. All results are based on a PAW/GGA/PBEsol VASP calculation.

**Atom**	***U* = 6 eV**	***U* = 7 eV**	***U* = 8 eV**	***U* = 8.77 eV** (used in Figure 11)	***U* = 9 eV**	***U* = 10 eV**
O	−1.80	−1.80	−1.81	−1.81	−1.81	−1.81
Mn	+1.80	+1.80	+1.81	+1.81	+1.81	+1.81

Table S4b. Löwdin Charges for MnO calculated with *U* parameters ranging from 6 to 10 eV. All results are based on a PAW/GGA/PBEsol VASP calculation.

**Atom**	***U* = 6 eV**	***U* = 7 eV**	***U* = 8 eV**	***U* = 8.77 eV** (used in Figure 11)	***U* = 9 eV**	***U* = 10 eV**
O	−1.25	−1.26	−1.27	−1.27	−1.28	−1.28
Mn	+1.25	+1.26	+1.27	+1.27	+1.28	+1.28

Table S5a. OS^{ai} for MnO_2 calculated with *U* parameters ranging from 5 to 10 eV. All results are based on a PAW/GGA/PBEsol VASP calculation.

**Atom**	***U* = 5 eV**	***U* = 6 eV**	***U* = 7.09 eV** (used in Figure 11)	***U* = 8 eV**	***U* = 9 eV**	***U* = 10 eV**
O	−1.46	−1.44	−1.42	−1.39	−1.36	−1.32
Mn	+2.92	+2.89	+2.84	+2.78	+2.72	+2.64

Table S5b. Löwdin Charges for MnO_2 calculated with *U* parameters ranging from 5 to 10 eV. All results are based on a PAW/GGA/PBEsol VASP calculation.

**Atom**	***U* = 5 eV**	***U* = 6 eV**	***U* = 7.09 eV** (used in Figure 11)	***U* = 8 eV**	***U* = 9 eV**	***U* = 10 eV**
O	−0.81	−0.83	−0.84	−0.84	−0.85	−0.86
Mn	+1.63	+1.65	+1.67	+1.69	+1.70	+1.72

Table S6. OS^{ai} for hydrogen peroxide, MnO and Mn_2O_7 calculated using the Bunge and the pbeVASPfit2015 basis set. All results are based on a PAW/GGA/PBEsol VASP calculation.

**Basis set**	**H_2O_2**		**MnO**		**Mn_2O_7**	
	**H**	**O**	**Mn**	**O**	**Mn**	**O**
Bunge	+0.79	−0.79	+1.81	−1.81	+4.21	−1.20
pbeVASPfit2015	+0.79	−0.79	+1.81	−1.81	+4.21	−1.20

Additional Charge Comparisons

To allow for a comparison of the OS^{ai} with density-based charge partitioning schemes, we also calculated Hirshfeld, Hirshfeld-I and Bader charges for H_2O_2, MnO, Mn_2O_7 and OF_2. The results are given in Table S7. The Hirshfeld and Hirshfeld-I charges were calculated directly using VASP whereas the Bader charges were determined in an additional step using the Critic2 program. The Hirshfeld charges indicate a rather small charge transfer, as expected for such charges. The iterative Hirshfeld method was designed to account for that, so we also included Hirshfeld-I results. Since they are described in literature[90] as to be found between Mulliken and Bader charges, it comes to no surprise that the calculated Hirshfeld-I charges are rather close to the Löwdin charges that are also numerically close to Mulliken charges. Bader charges indicate the largest transfer for all investigated systems. In all cases, however, (and especially for Mn_2O_7) they are clearly outperformed by the OS^{ai} that yield values even closer to the expected "on paper" oxidation numbers. The largest difference is found for Mn in Mn_2O_7 where the Bader charge is +1.86 compared to OS^{ai} = +4.21. The Bader charges are found in the same order of magnitude as the other partial charges here, which is unsurprising, since the Bader approach is a partitioning scheme designed to calculate partial charges but *not* oxidation numbers.

Table S7. OS^{ai} and Löwdin, Hirshfeld, Hirshfeld-I and Bader Charges for H_2O_2, MnO, Mn_2O_7, and OF_2. All results are based on a PAW/GGA/PBEsol VASP calculation.

	**H_2O_2**		**MnO**		**Mn_2O_7**		**OF_2**	
	**H**	**O**	**Mn**	**O**	**Mn**	**O**	**O**	**F**
OS^{ai}	+0.79	−0.79	+1.81	−1.81	+4.21	−1.20	+1.04	−0.52
Löwdin	+0.35	−0.35	+1.27	−1.27	+1.44	−0.41	+0.17	−0.09
Hirshfeld	+0.08	−0.08	+0.41	−0.41	+0.60	−0.17	+0.08	−0.04
Hirshfeld-I	+0.31	−0.31	+1.29	−1.29	+1.68	−0.48	+0.13	−0.07
Bader	+0.63	−0.63	+1.43	−1.43	+1.86	−0.53	+0.25	−0.12

Additional Information on the α Parameter

Before setting α to a constant value of 10, various test calculations were carried out. The results yield that there is a large range of α values where the results are unaffected, α merely smoothes out slight differences in orbital energies that would lead to an unnecessarily harsh assignment of bonding electrons, making the results scatter too much. The α value of 10 was chosen carefully since α needs to be as low as possible to allow for a smooth assignment of the bonding electrons while making sure that α is large enough to allow for a heterolytical assignment of all bonding electrons. To demonstrate what we mean by that, we have varied α from 0 to 100,000 for the case of hydrogen peroxide where covalency

is at play (Figure S2). The OS^{ai} of oxygen change from the value of the Löwdin Charge (−0.35) to the OS^{ai} value of −0.79 upon varying alpha from 0 to 3. After that, the values stay consistently the same for each oxygen atom. At an α value of 2000 that changes, and we are starting to observe a scatter of the OS^{ai} values because there are marginal differences in the orbital populations of the oxygen atoms. With the larger α value, the resulting OS^{ai} become more sensitive to that. When we vary α up to a value of 100,000, the distribution of oxygen OS^{ai} becomes even larger, leading to even more numerical noise. It needs to be mentioned that even at α = 100,000, however, the average of the oxygen OS^{ai} still remains at a value of −0.79. The OS^{ai} value of the H atoms has not been plotted here since it stays at a constant value of +0.79 from α = 3 up to 100,000. This means that we cannot use α to "fudge" our result, we can simply control the smoothness with which it is displayed and account for unnecessary harshness (i.e., overestimation of the bond polarization) when assigning bonding electrons.

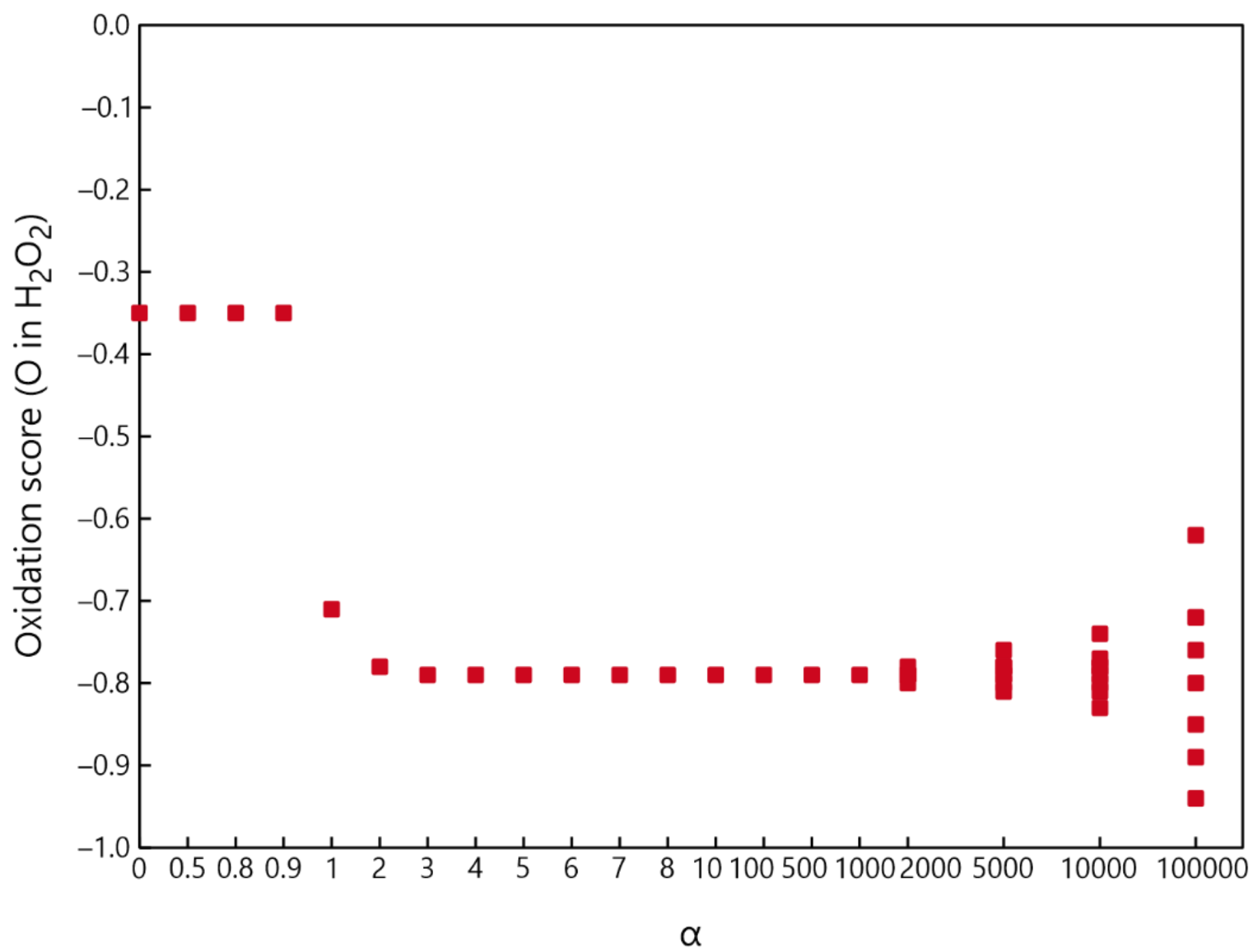

Figure S2. OS^{ai} of O in H_2O_2 upon variation of the α parameter from 0 to 100,000. The OS^{ai} of H are not shown here because they remain at a constant value of +0.79 starting from an α value of 3. Note how the value of the oxygen OS^{ai} remains constant from α values of 3 up to 1000. Above that, the average of the OS^{ai} does not change, the values simply scatter because the assignment of the bonding electrons becomes more sensitive to the most marginal differences in the individual orbital populations.
